# Twelve toll-like receptor (TLR) genes in the family Equidae – comparative genomics, selection and evolution

**DOI:** 10.1007/s11259-023-10245-4

**Published:** 2023-10-24

**Authors:** K. Stejskalova, E. Janova, P. Splichalova, J. Futas, J. Oppelt, R. Vodicka, P. Horin

**Affiliations:** 1 Department of Animal Genetics, Faculty of Veterinary Medicine, University of Veterinary Sciences Brno, Brno, 61242 Czech Republic; 2 RG Animal Immunogenomics, CEITEC VETUNI, University of Veterinary Sciences Brno, Brno, Czech Republic; 3 Zoo Prague, Prague, Czech Republic

**Keywords:** Toll-like receptor, Equid, Innate immunity, Transpecies haplotype sharing, Odd-toe ungulates

## Abstract

Toll-like receptors (TLRs) represent an important part of the innate immune system. While human and murine TLRs have been intensively studied, little is known about TLRs in non-model species. The order Perissodactyla comprises a variety of free-living and domesticated species exposed to different pathogens in different habitats and is therefore suitable for analyzing the diversity and evolution of immunity-related genes. We analyzed *TLR* genes in the order Perissodactyla with a focus on the family Equidae. Twelve *TLRs* were identified by bioinformatic analyses of online genomic resources; their sequences were confirmed in equids by genomic DNA re-sequencing of a panel of nine species. The expression of *TLR11* and *TLR12* was confirmed in the domestic horse by cDNA sequencing. Phylogenetic reconstruction of the TLR gene family in Perissodactyla identified six sub-families. TLR4 clustered together with TLR5; the TLR1-6-10 subfamily showed a high degree of sequence identity. The average estimated evolutionary divergence of all twelve TLRs studied was 0.3% among the Equidae; the most divergent CDS were those of *Equus caballus* and *Equus hemionus kulan* (1.34%) in the *TLR3*, and *Equus africanus somaliensis* and *Equus quagga antiquorum* (2.1%) in the TLR1 protein. In each *TLR* gene, there were haplotypes shared between equid species, most extensively in *TLR3* and *TLR9* CDS, and TLR6 amino acid sequence. All twelve *TLR* genes were under strong negative overall selection. Signatures of diversifying selection in specific codon sites were detected in all TLRs except TLR8. Differences in the selection patterns between virus-sensing and non-viral TLRs were observed.

**Supplementary Information:**

The online version contains supplementary material available at 10.1007/s11259-023-10245-4.

## Introduction

The emergence and evolution of infectious diseases result from a permanent confrontation between pathogens and hosts. Each of them use different strategies to survive. Pathogens often rely on their short generation interval and can rapidly change their surface antigens or target receptors. In higher organisms, the immune system has evolved over millions of years to cope with these changes. While the adaptive immune system responds to variable epitopes and compensates for pathogen variability via MHC class II, T- and B-cell receptors and antigen-specific antibodies, the innate, non-adaptive part of the immune system relies on evolutionarily older strategies. One of them is the recognition of conserved molecular patterns by various receptors (pattern recognition receptors, PRRs).

Toll-like receptors (TLRs) are representative examples of PRRs. They recognize patterns that are either associated with pathogens themselves (pathogen-associated molecular patterns, PAMPs) or released by damaged or dying cells (damage-associated molecular patterns, DAMPs). A typical TLR molecule contains three domains: an N-terminal pattern-recognizing outer part with multiple leucine-rich repeat domains (LRRs), a transmembrane domain, and an intracytoplasmic part with the toll/interleukin-1 receptor (TIR) domain responsible for intracellular signaling (reviewed e.g. in Behzadi et al. 2021). When activated, TLR signaling pathways elicit the production of type I interferons and inflammatory cytokines (Kawai and Akira 2011). Besides their role in innate immunity, TLRs can also modulate adaptive immune responses (Kumar 2022). Innate immune cells such as dendritic cells, macrophages and/or NK cells as well as epithelial and endothelial cells, but also T and B cells, express various TLRs. Mammalian TLRs 1, 2, 4, 5 and 6, which detect microbial cell components, are localized on the outer plasma membrane, while viral nucleic acid-sensing TLRs 3, 7, 8 and 9, as well as TLRs 10, 11, 12 and 13, are found in endosomes (as reviewed in Vijay 2018; Fitzgerald and Kagan 2020).

The Toll gene was first discovered in *Drosophila melanogaster* (Anderson et al. 1985), but evolutionary TLR prototypes have been identified in organisms pre-dating bilaterians, such as Cnidaria (reviewed in Brennan and Gilmore 2018). To date, 28 different TLRs have been identified in vertebrates, including 13 in mammals (Behzadi et al. 2021). Purifying selection appears to dominate the evolution of all vertebrate TLRs, but patterns of diversifying selection can be detected in specific codons concentrated in the ligand-binding domains (Liu et al. 2020). While negative selection preserves the essential functions of TLRs, diversifying selection helps TLRs cope with changes in the pathogen pressure. The spectrum of TLRs present in each species and the sites under selection thus reflect the history of species-specific host-pathogen interactions.

The functional importance of TLR gene polymorphisms, especially of single nucleotide polymorphisms (SNPs), is reflected in their associations with various types of diseases observed in multiple mammalian species. In humans, immunity-related gene polymorphisms are associated with increased susceptibility or resistance to various infectious agents, such as *Mycobacterium spp*., *Plasmodium spp*., and herpes viruses, as well as with an increased risk of cancer and autoimmune diseases such as Crohn’s disease and asthma (Mukherjee et al. 2019). In a meta-analysis, the *TLR4* 896 A/G polymorphism was associated with a higher risk of viral infections (Silva et al. 2022). In domestic animals, polymorphisms in various *TLR* genes have been associated with mastitis and other economically important traits in cattle (reviewed in Novák 2014). In horses, an association between a SNP in *TLR4* and West Nile virus infection has been reported (Stejskalova et al. 2019).

Compared to humans and model mammalian species, little is known about TLRs in non-model animals, such as domestic horses and the entire family Equidae. Despite their theoretical and practical importance, only fragmentary knowledge of their TLR genes consisting mostly of annotations in the current reference genome assemblies is available. As for their expression, *TLR1-10* mRNA has been identified in domestic horses (Uddin et al. 2016); *TLR 2* and *TLR5* expression was reported for the Damara zebra (Dugovich et al. 2019). Only sporadic reports focusing on the characterization of TLR9 have been published (Manuja et al. 2019; Smith et al. 2020). Although the Equidae family consists of a single genus, Equus (Price and Bininda-Emonds 2009), it includes a variety of free-living and domesticated species exposed to different pathogens in different habitats and is therefore suitable for analyzing the diversity and evolution of immunity-related genes (Janova et al. 2009).

The general aim of the study was to provide comprehensive factual information on the set of *TLR* genes in the entire family *Equidae* in the context of the entire order Perissodactyla. The specific objectives of this study were (i) to perform a comparative analysis of available genomic resources in terms of the presence, functionality, copy number, localization and genomic organization of *TLR* loci in all equid species (ii) to determine *TLR* coding sequences on a panel of equid species for all *TLR* loci identified, and (iii) to carry out evolutionary (phylogenetic) and selection analyses of individual *TLR* genes in the family Equidae.

## Materials and methods

### Study design

The aim of this study was to perform comparative analysis of *TLR* loci in available genomic resources of equid species in the context of the order Perissodactyla, to determine *TLR* coding sequences on an experimental set of equid species for all *TLR* loci identified, and to carry out evolutionary (phylogenetic) and selection analyses of individual *TLR* genes in the family Equidae and in the entire order Perissodactyla.

### Blood sample collection

Blood samples were collected from animals living in the ZOO Dvůr Králové and Prague ZOO, Czech Republic. Two (three where available) individuals representing the following nine species including four subspecies of the family Equidae were selected: Grevy’s zebra (*Equus grevyi*, EqGr); Mountain zebra (*Equus hartmannae*, EqHa); Plain zebras (*Equus quagga antiquorum -* EqQuAn, *Equus quagga boehmi -* EqQuBoe, *Equus quagga chapmani -* EqQuCh, *Equus quagga borensis* - EqQuBor); African wild ass (*Equus africanus somaliensis*, EqAfSo); donkey (*Equus asinus*, EqAs); Asian ass (*Equus kiang -* EqKi and *Equus hemionus kulan* - EqHeKu); domestic horse (*Equus caballus*, EqCa) and Przewalski’s horse (*Equus przewalskii*, EqPr). Since the taxonomic classification of zebras and donkeys is rather inconsistent, we followed the classification by ITIS (Integrated Taxonomic Information System; 10.5066/F7KH0KBK). Blood samples were stored at -20 °C until DNA extraction. All extracted DNAs were then used for sequencing of all *TLR* genes.

### Genomic resources

Four currently available equid reference genome assemblies and two non-reference assemblies, together with tapirs (*Tapirus indicus*, TaIn; *Tapirus terrestris*, TaTe), rhinoceroses (*Ceratotherium simum simum*, CeSi; *Diceros bicornis*, DiBi; *Rhinoceros unicornis*, RhUn; *Dicerorhinus sumatrensis*, DiSu), bovine (*Bos taurus*, BoTa), mouse (*Mus musculus*, MuMu) and human (*Homo sapiens sapiens*, HoSa) assemblies were searched for *TLR 1–13* (Table 1). In non-reference assemblies with no gene annotations available, the BLAST algorithm was used with *Equus caballus TLR* sequences as queries. Genomic sequences identified as *TLR* genes were then aligned for each individual gene. When multiple splice variants were present, the variant with a validated status was selected. In case all variants were only predicted, the one with coding sequence (CDS) length matching the length of the CDS of other species was chosen. Moreover, the NCBI nucleotide database was searched for equid *TLR* sequences using direct queries, BLASTn and tBLASTn algorithms. On the protein level, the UniProt database was searched. Data on domain structure and their localization within the gene and protein sequences were obtained from the NCBI GenBank and the UniProt database.

**Table 1:** Genome assemblies searched for *TLR 1–13* genes. * non-reference genomes

*Equus quagga*	UCLA_HA_Equagga_1.0 GCF_021613505.1	*Diceros bicornis minor*	mDicBic1.pat.decon GCA_020826835.1*
*Equus asinus*	ASM1607732v2 GCF_016077325.2	*Ceratotherium simum simum*	CerSimSim1.0 GCF_000283155.1
*Equus asinus*	ASM303372v1 GCA_003033725.1*	*Rhinoceros unicornis*	R_unicornis_scaffold_02 GCA_018403435.2*
*Equus caballus*	EquCab3.0 GCF_002863925.1	*Dicerorhinus sumatrensis*	ASM284483v1 GCA_002844835.1*
*Equss caballus*	Ajinai1.0* GCA_000696655.1	*Bos taurus*	ARS-UCD1.2 GCF_002263795.1
*Equus przewalskii*	Burgud GCF_000696695.1	*Mus musculus*	GRCm39 GCF_000001635.27
*Tapirus terrestris*	TapTer_v1_BIUU GCA_004025025.1*	*Homo sapiens*	GRCh38.p14 GCF_000001405.40
*Tapirus indicus*	TapInd_v1_BIUU GCA_004024905.1		

### DNA extraction

Two hundred microliters of whole blood were used for DNA extraction according to the manufacturer´s instructions (NucleoSpin Blood kit, Macherey-Nagel, Düren, Germany).

### Primer design and PCR

The genomic sequences of all equid *TLR* genes retrieved from the genomic resources were aligned in BioEdit (v7.2.5). Based on the *Equus caballus* EquCab3.0 sequence as a reference sequence, intron/exon boundaries and coding sequences (CDS) were determined for each *TLR* gene alignment. Conserved intronic regions surrounding the exon CDS were then identified and primer pairs for their amplification were designed by Primer-BLAST (https://www.ncbi.nlm.nih.gov/tools/primer-blast). PCR reactions were performed using EliZyme HS Robust MIX Red (Elisabeth Pharmacon, Brno, Czech Republic), 2x PCR BIO Ultra Mix (PCR BioSystems, London, United Kingdom) and Expand LongRange, dNTPack polymerase (Roche, Mannheim, Germany) according to the manufacturer’s instructions. The PCR reaction volume was 12.5 µl. Primer sequences and PCR protocols are given in Online Resource 1.

### *TLR* gene re-sequencing

A minority of PCR amplicons for *TLR4* were originally sequenced by Sanger sequencing (Macrogen, Seoul, South Corea); all other sequences were then obtained by next-generation sequencing of the same sample set. The Roche GS Junior 454 platform (Roche, Mannheim, Germany) as described in Bayerova et al. (2016) was used originally. However, the vast majority of the next-generation sequencing (NGS) was performed on the MiSeqTM System (Illumina, San Diego, California, USA). Sequencing libraries were prepared using the Nextera XT DNA Library Preparation Kit (Illumina, San Diego, California, USA). Amplicons from the same individual were tagged with the same index if there was no significant sequence similarity; otherwise, amplicons were indexed separately. Raw reads were checked in FastQC (v0.11.9) for quality and processed using Trimmomatic (v0.39). Reads were mapped to reference sequences by the BWA-MEM (v0.7.15) software. Alignments were checked using SAMtools (v1.4.1), GATK (v3.5) and Picard (v2.20.4). No more than 5% soft-clipping and 10% mismatches was allowed, and and a minimum read length of 70 nucleotides was required, for the final alignments using NGSUtils (v0.5.9) and BBMap (v38.58). NGS alignments were inspected in IGV software (v2.3.94) and further edited in BioEdit (v7.2.5). Consensus sequences were generated for each animal and each *TLR* gene. When potential heterozygous positions were identified, variable positions were verified by the SAMtools or GATK software; visual inspection and confirmation of the variable site was then done in the IGV browser. The confirmed heterozygous positions were replaced with IUPAC ambiguity code letters.

### cDNA sequencing

The expression of *TLR11* and *TLR12* in equine leukocytes was assessed by Sanger sequencing (Macrogen, Seoul, South Corea) and next-generation sequencing of equine cDNA amplified with primers designed based on the reference genome sequence and using Qiagen HotStarTaq Mix polymerase (Qiagen, Venlo, Netherlands) (see Online Resource 1 for primer sequence and reaction set up). Equine cDNA was prepared as described by Futas and Horin (2013).

### Bioinformatic sequence analyses

Alignments of genomic NGS-generated sequences were made for each *TLR* gene using BioEdit’s ClustalW multiple alignment algorithm and then trimmed according to the reference sequence to generate CDS alignments. Haplotypes were inferred using the PHASE algorithm in DnaSP (v6.12.03) and assigned to samples. Final alignments were then merged with previously retrieved alignments of GenBank sequences.

Amino acid sequences were inferred by translation of CDS following the standard genetic code. Variable nucleotide and amino acid sites were identified in MEGA (v11.0.13). The impact of amino acid changes on protein function and structure was evaluated at the Sorting Intolerant From Tolerant (SIFT, https://sift.bii.a-star.edu.sg/index.html) website.

Estimates of the evolutionary divergence between sequences were calculated in MEGA using a maximum composite likelihood model for nucleotide sequences and a Poisson correction model for protein sequences. For this purpose, tapirs, rhinoceroses, bovine, human and/or mouse sequences were included in the alignments (see Online Resource 2 for sequence IDs and Online Resource 3 for aligned sequences).

### Phylogenetic analysis

Combined alignments of both NGS-generated and GenBank retrieved sequences were evaluated for each *TLR* gene. The evolutionary history was reconstructed using maximum likelihood (ML) and the lowest BIC score nucleotide substitution model. The topology with the highest log-likelihood was always selected. Bovine and/or mouse sequences were used as outgroups to root the trees. Neighbor-joining trees (NJ) (uncorrected p-values, bootstrap consensus of 1000 replicates) were built as well. All calculations were performed in MEGA (v11.0.13).

### Selection analyses

All analyses were performed first for all perissodactyls and then for the Equidae separately. Merged alignments of both NGS-generated and GenBank retrieved sequences were evaluated. The codon-based Z-test of selection in MEGA software was used for the analysis averaging over all sequence pairs. First, the probability of rejecting the null hypothesis of strict neutrality (non-synonymous mutations frequency equals synonymous; dN = dS) was tested, followed by the probability of rejecting the null hypothesis in favor of one of the alternative hypotheses (dN > dS for diversifying selection, dN < dS for purifying selection). The variance of the difference was calculated using the bootstrap method (1000 replicates). Analyses were performed using the Kumar method. Pervasive site-specific selection was evaluated by three methods (FEL, FUBAR, SLAC; performed by Datamonkey.org); selected amino acid sites (SAAS) where *p* was ≤ 0.05 or at *p ≤* 0.1 but confirmed by two methods were considered to be under selection. Episodic site-specific diversifying selection was tested by MEME (Datamonkey.org); here, *p* values ≤ 0.05 were considered significant. Fisher’s exact probability test (two-tailed) was used to compare rates of SAAS in different groups of TLRs.

## Results

### *In silico* analysis of *TLR* genes in equids

Twelve functional *TLR* genes (*TLR1-12*) were identified in all the equid genomes analyzed. All coding sequences retrieved *in silico* are provided in Online Resource 3. The GenBank search did not yield any additional results for *TLR* sequences of equid origin beyond those derived by prediction from the assemblies. The major features of the genomic organization of *TLRs* in equids, including chromosomal locations, gene and protein length, and exon counts, are summarized in Table 2. Multiple splice variants were predicted for some of the *TLR* genes analyzed, with a maximum of 10 variants for *TLR8* in *Equus caballus* and *Equus asinus*.

Although in general the *TLR* sequences were conserved across the species analyzed, some exceptions were observed. The computer-predicted, but not validated, CDS of *TLR2* in *Equus przewalskii* and *TLR9* in *Equus quagga* differed greatly in length from the rest of equids. Therefore, they were excluded from further bioinformatic analyses.

Twelve functional *TLR* genes (*1–12*) were also identified in available genomes of other odd-toed ungulates, namely tapirs and rhinoceroses. The predicted CDS of *TLR2* based on BLAST hits in *Tapirus terrestris* as well as *TLR2, 6* and *11* in *Rhinoceros unicornis* were excluded from further analyses due to incomplete sequences and/or to frameshifts with multiple stop codons.

**Table 2:** Genomic organization of *TLRs* in equids based on reference genomes. More variants are shown due to multiple splice variants predicted for some genes. The length of the variant chosen for analysis is in bold. Ec – *Equus caballus*, Ep – *Equus przewalskii*, Ea – *Equus asinus*, Eq – *Equus quagga*, Un – unknown

TLR	chromosome				gene length (bp)				exons spanning CDS				predicted protein length (AA)			
	*Ec*	*Ep*	*Ea*	*Eq*	*Ec*	*Ep*	*Ea*	*Eq*	*Ec*	*Ep*	*Ea*	*Eq*	*Ec*	*Ep*	*Ea*	*Eq*
**1**	3	Un	3	3	4901	4929	62,550	62,160	1	1	1	1	**786**	786	786	786
**2**	2	Un	3	3	24,873	5382	11,898	11,181	1–2	1	1	1	**784**	742	784	784
**3**	27	Un	27	22	16,921	16,099	15,712	16,806	4–5	4	4–5	4–5	**904**, 920	904	904, 908	904, 908
**4**	25	Un	10	1	10,377	10,769	10,439	10,652	3	3	1–3	1–3	**843**	843	752, 843	752, 843
**5**	30	Un	30	12	26,703	26,367	16,979	46,432	1	1–2	1	1	**859**	859, 867	859	859
**6**	3	Un	3	3	30,825	28,168	29,225	24,317	1–2	1–2	1–2	1–2	**796**, 928	796, 927	796, 822, 927	796, 822
**7**	X	Un	Y	10	24,192	24,199	24,269	24,287	2	2	1–2	2	**1050**, 1054	1050, 1054	1049, 1050, 1054	1050, 1054
**8**	X	Un	Y	10	19,102	7032	26,867	16,711	1–2	1	1–2	1–2	928, **1038**, 1048	1038	928, 1038, 1041, 1048	928, 1038, 1048
**9**	16	Un	21	1	16,342	4831	4361	4773	2–5	3	2	3	**1031**, 1324	1031	1031	1074
**10**	3	Un	3	3	26,157	26,082	21,112	4743	1	1	1	1	**811**	811	811	811
**11**	1	Un	2	2	7959	5894	6485	2963	1	1	1	1	**923**	923	923	923
**12**	2	Un	5	5	3565	4232	4301	3161	1–2	1	1	1	**908**, 949	908	908	908

**Fig. 1:**
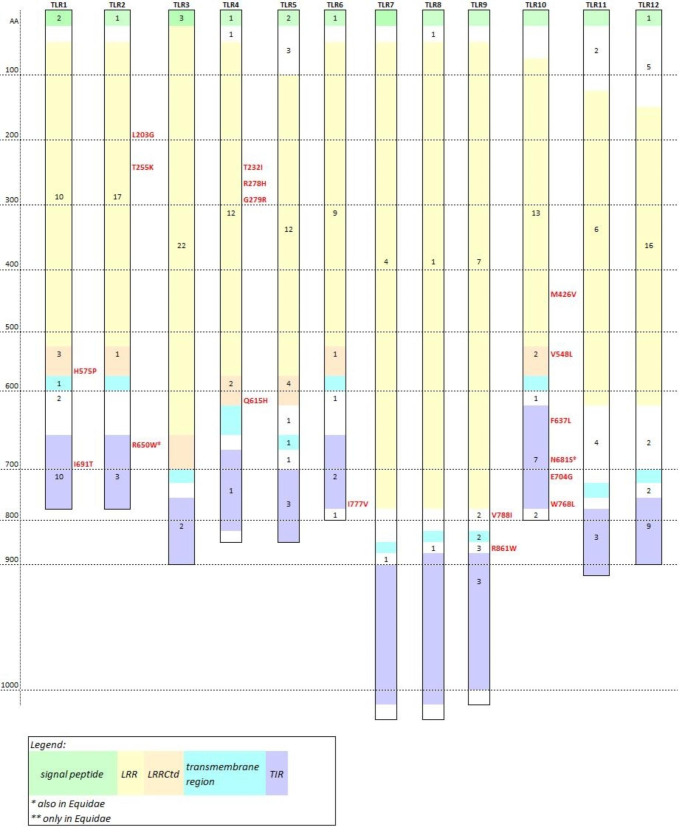
A graphic overview of known and/or predicted domain organization of TLR1-12 in *Equus caballus.* Some domain positions were inferred from human and murine data (see Online Resource 4 for details). The LRR region, shown as a single block for simplicity, represents a region where numerous LRR domains (typically 10–30 in number) occur at different spacing. The number inside each domain indicates the sum of variable amino acid (AA) sites identified in translated alignments of NGS and GenBank retrieved sequences in the family Equidae. AA changes which may affect the protein function (according to SIFT) are in red. *This variation was only present in GenBank data; LRRCtd – Leucine-rich repeat C-terminal domain

All equid *TLR* genes and predicted proteins examined shared common features with their mammalian orthologues. The overview of TLR1-12 protein structure, i.e. the position of the signal peptide, of the LRR domain region and the LRR-C-terminal domain, and of the transmembrane region and the TIR domain, is shown in Fig. 1. Details including the exact locations of the respective domains are provided in Supplementary Table 1 in Online Resource 4.

In terms of *TLR* gene expression, mRNAs and derived CDS have so far been mostly predicted only *in silico*, and thus have a provisional status. A validated status has been assigned to the *TLR1, 4, 8, 9* reference sequences of the domestic horse. According to the UniProt database, there is evidence of gene transcription for horse *TLRs 2–4* and *7–10*, and evidence for translation for *TLR9* in domestic horses and donkeys. However, the expression of equine TLR1-10 has been confirmed as well (Astakhova et al. 2009; Uddin et al. 2016; Tarlinton et al. 2016). Dugovich et al. (2019), and Smith et al. (2020) confirmed the expression of *TLR2, 5* and *9* in *Equus quagga spp*.

### Re-sequencing of equid *TLR* genes

Coding sequences of all 12 *TLR* genes were obtained for all 12 species and subspecies included in the experimental panel. All sequences were submitted to GenBank; accession numbers are provided in Online Resource 5, while complete alignments are provided in Online Resource 3. On average, 1.69% of all CDS nucleotide positions and 2.27% of amino acid positions were variable between the species of the family. The least variable was the *TLR8* sequence (0.96/0.29%); the highest variability was observed in *TLR12* (2.46/3.85%). The numbers of unique haplotypes ranged from 18 in *TLR7* and *TLR8* to 32 in *TLR12*. See Table 3 for detailed numbers.

**Table 3:** Frequency of variable nucleotide and amino acid sites identified in combined alignments of *TLR1-12* sequences obtained by NGS and from GenBank in the family Equidae. Haplotypes inferred from NGS data along with additional unique sequences from GenBank were added together in the unique sequences column

TLR	cds sequences evaluated	unique cds sequences identified	cds length	variable cds sites	variable amino acid sites
**1**	31	25	2358	40 (1,70%)	28 (3,56%)
**2**	32	26	2352	42 (1,79%)	22 (2,81%)
**3**	39	29	2712	61 (2,25%)	27 (2,99%)
**4**	30	23	2529	40 (1,58%)	17 (2,02%)
**5**	34	26	2577	51 (1,98%)	27 (3,14%)
**6**	32	23	2388	32 (1,34%)	15 (1,88%)
**7**	26	18	3150	32 (1,02%)	5 (0,48%)
**8**	28	18	3114	30 (0,96%)	3 (0,29%)
**9**	34	24	3093	61 (1,97%)	16 (1,55%)
**10**	29	21	2433	42 (1,73%)	25 (3,08%)
**11**	31	23	2769	43 (1,55%)	15 (1,63%)
**12**	39	32	2724	67 (2,46%)	35 (3,85%)

### *In silico* inferred amino acid TLR sequences

Altogether, 208 variable amino acid sites were identified in TLR1-12 in the Equidae. Online Resource 4, Suppplementary Table 2, provides the complete list of variable sites detected and their localization within the respective protein; a brief summary is presented in Fig. 1. Eighteen variable amino acid sites were identified by SIFT as potentially affecting protein function (SIFT scores for all sites are provided in Online Resource 4). Six of them were identified in TLR10, four of them in the TIR domain. A rather high proportion of variable AA sites located in the TIR domain was observed in TLR1, 10 and 12.

### cDNA sequencing

Sanger and NGS sequencing showed that equine *TLR11* and *TLR12* are transcribed genes and confirmed their expression in equine white blood cells. The sequences retrieved (provided in the Online Resource 3) matched the predicted coding sequences for these two genes. Both sequences were submitted to GenBank under accession numbers OQ971889 (TLR11) and OQ971888 (TLR12).

### Interspecific comparisons and trans-species allele sharing

The average estimated CDS evolutionary divergence of all twelve TLRs studied was 0.3% among the Equidae, with the lowest value for *TLR7* (0.13%), and the highest value for *TLR3* (0.5%). The most divergent sequences were those of *Equus caballus* and *Equus hemionus kulan* (1.34%) in *TLR3*. On the other hand, in each TLR gene there were identical haplotypes shared between species (see Table 4; all shared haplotypes and species combinations can be found in Online Resource 6 and 7). While only one out of 26 haplotypes was shared in *TLR2*, five haplotype alleles were shared in *TLR3* (out of 29) and in *TLR9* (out of 24). At the amino acid level, the number of AA sequences shared by at least two species ranged from one (in TLR8) to five (in TLR3 and TLR6). The only AA sequence shared in TLR8 covered the widest range of species, as it was detected in all equids except *Equus quagga* and *Equus hemionus kulan.*

**Table 4:** A summary of trans-species allele sharing observed in the family Equidae in TLR1-12. Species sharing the common haplotype (nucleotide coding sequence) are shown in bold, while species sharing AA sequences are in plain text. Some species shared more than one allele. EqGr – *Equus grevyi;* EqHa – *Equus hartmannae;* EqQu – *Equus quagga;* EqAfSo – *Equus africanus somaliensis;* EqAs – *Equus asinus;* EqKi – *Equus kiang;* EqHeKu – *Equus hemionus kulan;* EqCa – *Equus caballus;* EqPr – *Equus przewalskii*

	CDS alleles shared	AA sequences shared	species sharing the common allele
TLR1	2	3	**EqCa-EqPr** **EqCa-EqPr-** EqQu EqAs-EqAfSo
TLR2	1	2	**EqCa-EqPr** EqAs-EqAfSo
TLR3	5	5	**EqGr-EqQu** **EqHa-EqQu** **EqAs-EqAfSo** **EqCa-EqPr** 2x
TLR4	3	4	**EqAs-EqAfSo** **EqHeKu-EqKi**-EqGr-EqQu **EqCa-EqPr** EqCa-EqPr
TLR5	2	2	**EqAs-EqCa-EqPr** **EqAs-EqAfSo**
TLR6	4	5	**EqGr-EqQu**-EqAfSo **EqCa-EqPr** 2x EqCa-EqPr EqHeKu-EqKi
TLR7	4	2	**EqAs-EqAfSo-EqHa**-EqHeKu-EqKi **EqHeKu-EgKi** **EqCa-EqPr** 2x
TLR8	4	1	**EqAs-EqAfSo**-EqGr-EqHa-EqKi-EqCa-EqPr **EqHa-EqKi** **EqCa-EqPr** 2x
TLR9	5	3	**EqGr-EqQu**-EqHa **EqAs-EqAfSo** 2x **EqCa-EqPr** 2x
TLR10	4	4	**EqGr-EqQu** **EqAs-EqAfSo** **EqCa-EqPr** 2x
TLR11	2	2	**EqCa-EqAs** 2x EqHeKu-EqKi
TLR12	2	4	**EqAs-EqCa-EqPr** **EqCa-EqPr** EqCa-EqPr EqHeKu-EqKi

When comparing the nucleotide sequences of equids with other odd-toed ungulates, the average divergence between equids and tapirs was 6.69%, while it was 6.89% between equids and rhinoceroses. The least divergent were the sequences of *TLR6*, while the most different were *TLR4* in tapirs (9.86%) and *TLR12* in rhinoceroses (10.22%). No haplotype sharing was observed between equids and tapirs or rhinoceroses. The average divergence from bovine, human and murine sequences were 14.62%, 13.94% and 27.79%, respectively. In these species, the sequence of *TLR8* was the most different from equids. Detailed interspecific comparison is provided in Online Resource 6.

At the protein level, the average estimated amino acid sequence divergence of all twelve TLRs was 0.44% among the Equidae; the divergence was lowest in TLR8 (0.08%) and highest in TLR1 (0.72%). The most different were *Equus africanus somaliensis* and *Equus quagga antiquorum* (2.1%) in TLR1. When comparing equids with other perissodactyls and bovine amino acid sequences, the most different were sequences of TLR4. Compared to humans, the most different was TLR8; compared to mice, the most different was TLR12. The estimated average divergence from equids to tapirs was 10.15%, while to rhinoceroses it was 10.53%. The average divergence from bovine, human and murine sequences were 19.69%, 19.95% and 32.6%, respectively. Divergence matrices for all species combinations on both the CDS and protein levels are provided in Online Resource 6 and 7, respectively.

### Phylogenetic analyses

The inferred phylogenetic history represented by the constructed trees was generally consistent with the current taxonomy of the Perissodactyla order and the family Equidae, where three basic clades can be distinguished: zebras, asses and horses. However, occasional deviations from neutrality were observed in individual trees, although some lineages were only weakly supported. For the sake of better readability of the main text, all Neighbor-joining (NJ) and maximum-likelihood (ML) trees for TLR1-12 in the Perissodactyla are provided only as Supplementary Figs. 1–24 (Online Resource 8).

**Fig. 2:**
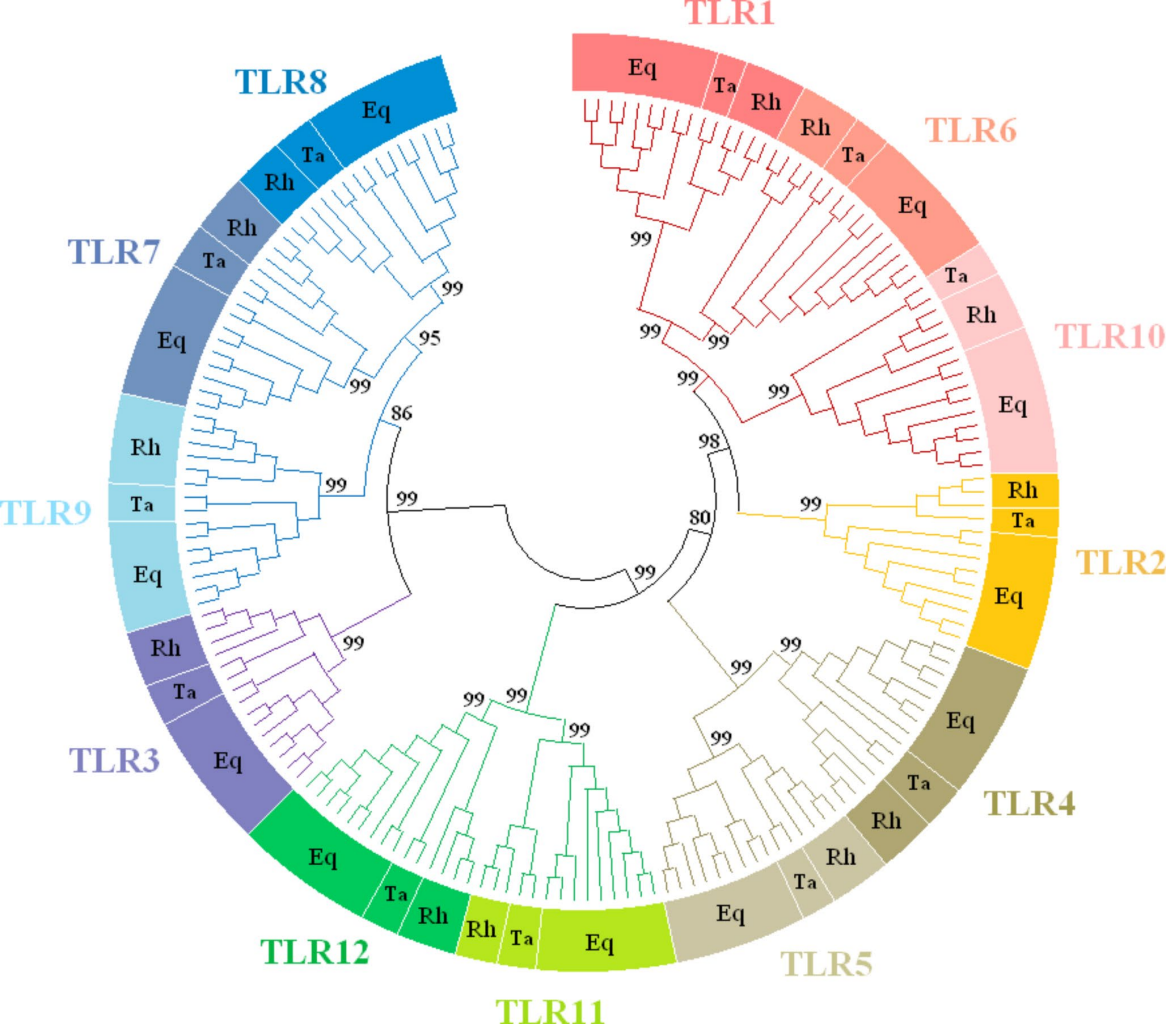
The phylogeny of TLR1-12 in the Perissodactyla inferred by the Maximum Likelihood method and the General Time Reversible model. The tree with the highest log likelihood (-63670.76) is shown. This analysis involved 177 nucleotide sequences, one representative sequence per species for each TLR gene. The percentage of trees (1000 bootstraps) in which the associated taxa clustered together is shown next to the branches. Eq – equids, Ta – tapirs, Rh – rhinoceroses

Figure 2 shows an unrooted ML tree with twelve TLR genes clustering into six families: the family of TLR1-6-10 with the single member family of TLR2 branching next to it; the two-member families of TLR4, 5 and TLR11,12; then TLR3 which clustered by itself but in proximity to the family comprising TLR 7, 8 and 9.

### Selection analyses

The effects of selection acting on entire genes are summarized in Table 5. For the whole order Perissodactyla, all twelve *TLR* genes were under very strong negative selection. For the Equidae alone, six genes - *TLR 3, 4, 7, 8, 9* and *11* - showed a deviation from neutral evolution at the p < 0.05 significance level. Strong negative selection was also confirmed in these six genes, most evidently in *TLR9*. Evidence of diversifying selection acting on whole genes was detected neither in perissodactyls nor in equids.

**Table 5:** Codon-based Z-test of selection for CDS alignments of *TLR1-12* where H_0_: dN = dS. H_0_ – null hypothesis; H_A_ – alternative hypothesis; dS and dN are the frequencies of synonymous and nonsynonymous substitutions per site, respectively. Significant values are in bold

Perissodactyla					Equidae			
*p* (H_A_:dN ≠ dS)	neutral evolution	*p* (H_A_:dN < dS)	negative selection	**TLR**	*p* (H_A_:dN ≠ dS)	neutral evolution	*p* (H_A_:dN < dS)	negative selection
**0.00011**	-3.98876	**0.00008**	3.90152	**1**	0.32143	-0.99564	0.17850	0.92467
**0.00001**	-4.55638	**2.89e-6**	4.74653	**2**	0.11087	-1.60616	0.05656	1.59603
**< 1.e-10**	-8.71969	**< 1.e-10**	8.79839	**3**	**0.00233**	-3.11084	**0.00092**	3.18493
**0.00144**	-3.26213	**0.00053**	3.35845	**4**	**0.03352**	-2.15049	**0.01640**	2.15939s
**7.61e-7**	-5.2198	**4.98e-7**	5.15836	**5**	0.13774	-1.49422	0.06722	0.150697
**0.00002**	-4.5079	**2.97e-6**	4.73943	**6**	0.08093	-1.76017	0.05071	1.65072
**< 1.e-10**	-7.24292	**< 1.e-10**	7.31086	**7**	**0.00164**	-3.22227	**0.00079**	3.23308
**< 1.e-10**	-8.69243	**< 1.e-10**	9.127	**8**	**0.00019**	-3.85838	**0.00009**	3.88086
**< 1.e-10**	-7.67791	**< 1.e-10**	7.93906	**9**	**0.00004**	-4.29579	**0.00002**	4.25242
**3.51e-7**	-5.39397	**1.85e-7**	5.19027	**10**	0.1803	-1.34767	0.09369	1.32595
**1.8e-9**	-6.51906	**4e-10**	6.67298	**11**	**0.00109**	-3.34691	**0.00068**	3.27972
**3.35e-6**	-4.8762	**9.36e-6**	4.45812	**12**	0.13917	-1.48876	0.07761	1.43030

**Fig. 3:**
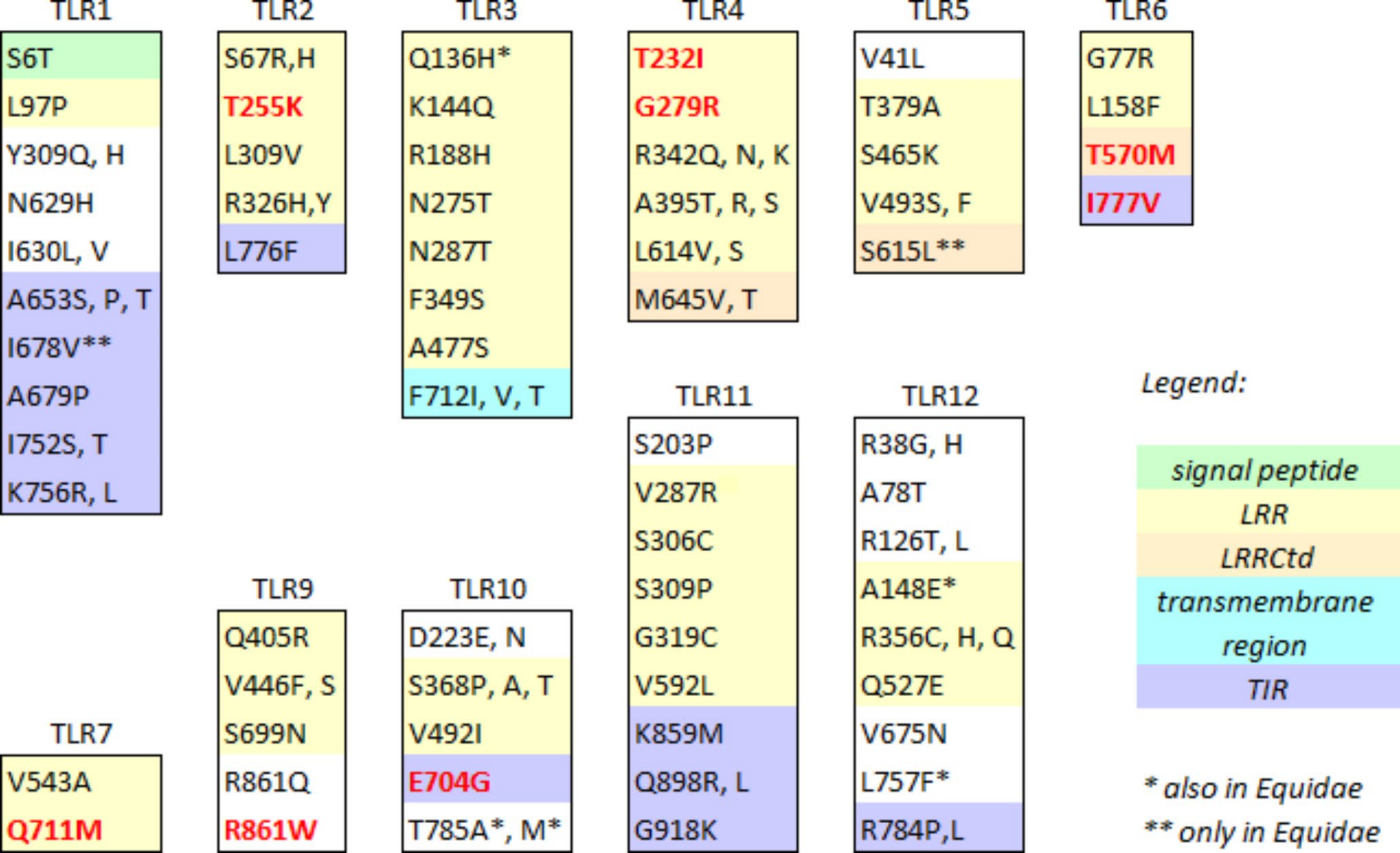
Positively selected amino acid sites (PSS) in TLR1-12 in the Perissodactyla. Site positions correspond to the sequence of *Equus caballus*. AA changes which may affect protein function (according to SIFT) are in red

However, individual selected amino acid sites (SAAS) were observed both in the Perissodactyla and in the Equidae. The full list of SAAS, including p-values, along with a summary table (Supplementary Table 3), are provided in Online Resource 9. Purifying selection clearly predominated, as only 65 of the 615 SAAS detected were under diversifying selection in the Perissodactyla (0.61% of all sites analyzed), and 8 out of 103 in the Equidae (0.06% of all sites analyzed). An overview of the numbers of sites under episodic and pervasive site-specific positive selection identified in the Perissodactyla within each TLR is provided in Table 6. Figure 3 shows amino acid changes at all PSS, their distribution within the domains of respective proteins and SIFT prediction of the change impact. The majority of PSS (positively selected sites) were located in the LRRs and LRR-Ct domains (60% in the Perissodactyla, 50% in the Equidae), while 18.5% (16.7% in the Equidae) were located in the TIR domain. Interestingly, there were four PSS located in the TIR domain of TLR1 in the Perissodactyla, in contrast to just one in the LRR region.

**Table 6:** Positively selected amino acid sites in TLR1-12 in the Perissodactyla. Only significant results are shown: *p ≤ 0.05* or 2x *p ≤ 0.1* for pervasive selection (FEL and FUBAR methods, SLAC results were insignificant for all sites tested), *p ≤ 0.05* for episodic selection (MEME). Asterix marks PSS significant also (*) or only(**) in the Equidae-only alignment. ^*#*^ Behzadi et al. (2021), Andrade et al. (2013), Hatai et al. (2016)

TLR	Codon site	FEL	FUBAR	MEME	Type of positive selection	Known TLR ligands^#^
1	6			0.00	Episodic	Bacterial lipopeptides
	97	0.056	0.07		Pervasive	
	309		0.034		Pervasive	
	629		0.045		Pervasive	
	630		0.048		Pervasive	
	653	0.098	0.044		Pervasive	
	678**		0.037		Pervasive	
	679			0.00	Episodic	
	752		0.028		Pervasive	
	756	0.061	0.035		Pervasive	
2	67		0.05		Pervasive	Bacterial lipids, fungal zymosan, mannan, HSP70
	255			0.02	Episodic	
	309	0.089	0.032		Pervasive	
	326		0.045		Pervasive	
	776	0.091	0.058		Pervasive	
3	136*			0.02	Episodic	Viral dsRNA, endogenous RNAs
	144			0.01	Episodic	
	188		0.041		Pervasive	
	275			0.05	Pervasive	
	287			0.05	Pervasive	
	349			0.02	Pervasive	
	477			0.02	Pervasive	
	712	0.032	0.009	0.05	Pervasive/episodic	
4	232			0.05	Episodic	LPS, endogenous DAMPs
	279			0.05	Episodic	
	342		0.045		Pervasive	
	395	0.0950	0.033		Pervasive	
	614	0.0673	0.076		Pervasive	
	645	0.0924	0.046		Pervasive	
5	41			0.03	Episodic	Bacterial flagellin
	379			0.05	Episodic	
	465			0.05	Episodic	
	493	0.019	0.045		Pervasive	
	615**		0.049		Pervasive	
6	77			0.05	Episodic	Bacterial lipoproteins, endogenous DAMPs
	158	0.084	0.063		Pervasive	
	570			0.04	Episodic	
	777			0.03	Episodic	
7	543			0.03	Episodic	ssRNA, immune complexes
	711			0.03	Episodic	
9	405			0.04	Episodic	CpG DNA, DNA/RNA hybrids
	446		0.033		Pervasive	
	699			0.03	Episodic	
	861		0.038	0.02	Pervasive	
10	223			0.02	Episodic	Lipoproteins, viral glycoproteins, dsRNA
	368		0.031		Pervasive	
	492		0.043		Pervasive	
	704			0.02	Episodic	
	785*			0.05	Episodic	
11	203			0.05	Episodic	Bacterial flagellin, *T. gondii* profilin
	287			0.00	Episodic	
	306			0.04	Episodic	
	309			0.05	Episodic	
	319			0.04	Episodic	
	592			0.03	Episodic	
	859			0.02	Episodic	
	898	0.072	0.085		Pervasive	
	918			0.01	Episodic	
12	38			0.04	Episodic	Bacterial flagellin, *T. gondii* profilin
	78			0.02	Episodic	
	126	0.098	0.066		Pervasive	
	148*			0.01	Episodic	
	356	0.09	0.07		Pervasive	
	527			0.05	Episodic	
	675			0.02	Episodic	
	757*			0.02	Episodic	
	784	0.061	0.054		Pervasive	

### Comparisons of selected amino acid sites – interspecific differences

PSS identified in the Equidae and the Perissodactyla were compared to PSS previously described in vertebrates (Liu et al. 2020). Matches were found for the following PSS: TLR4-R342, A395; TLR5- V493; TLR9- V446, S699; TLR11- V287; TLR12- A78, A148, V675 (*Equus caballus* site positions). In comparison with the solely mammalian PSS identified by Areal et al. (2011), four shared PSS were found: TLR3-F712; TLR4-A395, L614; TLR10-V492.

### Comparisons of selected amino acid sites – “Viral” vs. “Non-viral” TLRs

Comparisons between numbers of PSS and negatively selected sites (NSS) among non-viral (TLR1, 2, 4, 5, 6, 11, 12) and viral TLRs (TLR 3, 7, 8, 9) in the Perissodactyla showed more NSS and fewer PSS in the viral group than in the non-viral group (Fisher’s exact test, *p* = 2.5e-7). Separate comparisons of PSS and NSS rates within the LRR domains and the TIR domains also showed a significant difference between the viral and non-viral group (*p* = 0.00062 for LRR, *p* = 0.00061 for TIR domains). No significant difference between non-viral and viral TLR groups was found for the Equidae alone.

## Discussion

*TLR* genes and their evolution have been studied in various vertebrate groups, including several mammalian families and model mammalian species, such as humans and mice (Liu et al. 2020). This study brings comprehensive information on the genomic organization and evolution of *TLR* genes in the family Equidae in the evolutionary context of the order Perissodactyla.

Both *in silico* analyses of WGS from GenBank and the resequencing of genomic DNA amplicons showed that equids as well as other perissodactyls (rhinoceroses and tapirs) possess 12 *TLR* genes. They are highly conservative in terms of their nucleotide sequences and the structure of their putative protein products. Based on the data obtained, it may be assumed that they play similar roles in immunity as in other mammalian species. In the mouse genome, *TLRs 1–9* and *TLRs 11–13* have been identified, while *TLR10* is considered missing (Kawai and Akira 2011). In the human genome, ten *TLR* genes (*1–10*) and a *TLR12* pseudogene have been annotated so far. In the current reference genome version of cattle (ARS-UCD1.2), only *TLRs 2–10* have been annotated. However, the expression of the TLR1-6-10 family was confirmed and the genomic organization of the corresponding chromosome region was determined (Opsal et al. 2006). In agreement with this finding, the UniProt database contains data on bovine *TLR1* identified at transcript level (ACH92575.1).

Unlike humans but similarly to mice, the genomes of all species analyzed here contained the *TLR11* and *TLR12* genes. Both of these genes can be found also in Rodentia, Lagomorpha, several Chiroptera species, and also in the Elephantidae. A *TLR12*, but not a *TLR11* sequence has been annotated in most of the Cetaceans’ genomes as a pseudogene. Neither of the two genes can be found in genomes of other Cetartiodactyla members, such as Bovidae or Suidae. TLR11 recognizes bacterial flagellin (Mathur et al. 2012) and protozoan profilin (Yarovinski et al. 2005). The latter is also the main ligand of TLR12, and TLR11/12 heterodimers play important roles in the resistance of mice to *Toxoplasma gondii* (Koblansky et al. 2013; Andrade et al. 2013). As rodents are the major intermediate hosts for this parasite, the rodent immune system is adapted to better cope with *T. gondii* infection (Gazzineli et al., 2014). Possible role(s) of TLR11/12 in equine immune mechanisms involved in responses to *T. gondii* infection are yet to be elucidated. While clinical toxoplasmosis in horses is extremely rare (Kimble et al. 2021), the seroprevalence among equids is high, as many serological studies have detected antibodies against *T. gondii* in both domestic and wild equids (reviewed in Dubey et al. 2020). Here, we have shown that both *TLR11* and *TLR12* are transcribed in *Equus caballus* white blood cells. This is consistent with the expression pattern of these two genes described in mice (Koblansky et al. 2013). The presence of potentially functional genes *TLR11* and *TLR12* is a special feature of the *Equidae* that merits further attention in the context of their immune mechanisms and resilience/susceptibiliy to diseases.

According to the current annotation of the donkey genome (ASM1607732v2), both *TLR7* and *TLR8* are located on the Y chromosome in *Equus asinus.* This is in disagreement with the annotations of *TLR7* and *TLR8* genes in other mammalian species as well as with our findings. In most mammals – including humans, rodents, cattle and horses – these two genes are located on the X chromosome; in *Equus quagga spp.* (UCLA_HA_Equagga_1.0) they are currently annotated on chromosome 10. Both of the two *Equus asinus* samples we examined were heterozygous for two different TLR7 alleles, and both of the two African wild ass (*Equus africanus somaliensis*) samples were heterozygous for two different TLR8 alleles. We have checked the genes flanking *TLR7* and *TLR8* in this *Equus asinus* genome assembly, and they Blast-mapped in the vicinity of *TLR7* and *TLR8* on the X chromosome in the horse reference genome. Therefore, the localization of the donkey *TLR7* and *TLR8* genes on the X chromosome is likely to be an accurate assumption. The assembly of this part of the donkey genome thus seems to be incorrect due to the limitations of the short-read technique, but there is currently no WGS based on long reads that could confirm this assumption. Similarly, it seems that the localization of *TLR7* and *TLR8* on chromosome 10 in *Equus quagga spp.* might be due to an incorrect assembly of the genome of this species.

*TLRs 7* and *8* show the lowest variability across the Equidae species. The low number of variable nucleotide positions combined with high rate of synonymous substitutions result in a very low number of variable amino acid sites. In *TLR8*, 90% of nucleotide substitutions were synonymous: 18 CDS haplotypes created only 4 amino acid alleles. Two of them were specific to *Equus quagga spp.*, one to *Equus hemionus kulan*, and the remaining one was shared across the remaining equids. This is consistent with the high degree of conservation of the *TLR7* and *TLR8* genes previously described in mammals (Khan et al. 2019), except for lagomorphs (Neves et al. 2022). Interestingly, the highest variability was observed for *TLR12*, a gene missing in the genomes of several mammalian species (Behzadi et al. 2021), with a single currently known ligand, the *T. gondii* profilin. TLR11/12 heterodimers are required to elicit a response to profilin (Andrade et al. 2013); *TLR11* showed a low degree of polymorphism in equids.

The phylogenetic analysis of *TLR* genes revealed six *TLR* gene clusters in the Perissodactyla. The clusters generally matched the *TLR* families recognized in vertebrates : TLR1-6-10,2; TLR3; TLR4; TLR5; TLR7-9; TLR11-23 (Roach et al. 2005; Liu et al. 2020). In our tree, *TLR4*, which clustered together with *TLR5* with very high branch support (99%), represents an exception. According to Roach et al. (2005) and Liu et al. (2020), *TLR4* and *TLR5* are single members of separate TLR families. It is not clear whether the different position of the *TLR4* in perissodactyls is due to the overall conservation and lesser differentiation of the entire Equidae family.

A conserved synteny of three paralogue genes, *TLRs 1-6-10*, was observed. This gene family arose by successive tandem duplications of an ancestral gene. In mammals, *TLR10* emerged first, followed by *TLR1* and *TLR6* (Roach et al. 2005). In agreement with Kruithof et al. (2007), we observed a very high degree of sequence identity in a region of 300 amino acids (approx. 440–740) in the C-terminus of equid TLR1 and TLR6, which could be due to a gene conversion. There was no such a region of similarity found between TLR1 and TLR10, or TLR6 and TLR10. *TLR10* diverged from the common ancestor much earlier than *TLR1* and *TLR6*. Based on currently known TLR ligands (reviewed in Behzadi et al. (2021), the recognition of bacterial lipopeptides remained preserved in all three members of the *TLR-1* gene family; TLR10 gained additionally the ability to recognize viral motifs, which may explain its lower sequence similarity to TLR1 and TLR6.

Phylogenetic trees reconstructed for individual TLRs in the Perissodactyla showed that in agreement with the current taxonomy, rhinoceroses, tapirs and equids always formed distinct groups. Within the Equidae group, the clustering of species was less distinct. According to the available mitochondrial and nuclear gene analysis, caballines, asian asses, african asses and zebra clades can be distinguished (Steiner and Ryder 2011). However, despite the general conservation of the *TLR* genes, a clear separation between zebras and asses was not always observed (Online Resource 8). As immune-related genes, *TLR* genes are subject to various selective pressures, reflecting the history of host-pathogen interactions. A dynamic balance between diversifying and balancing selection then drives allelic variation within and between species to cope with changes in PAMPs (Minias and Vinkler 2022). The deviations from the zoological taxonomy observed in the phylogenetic trees may be interpreted as deviations from neutrality, which is in agreement with the general findings of diversifying selection reported for *TLR* genes in different mammalian families (Ghosh et al. 2022; Darfour-Oduro et al. 2016).

The idea of the functional importance of some of the observed polymorphic variants is also supported by the findings of trans-species allele sharing and the presence of PSS in equids. In general, inferred *TLR* allelic haplotypes were shared mostly within equid clades (e.g. *E. caballus-E. przewalskii*), but for *TLR 1, 4, 5, 6, 7, 8, 11, 12*, at least one allele was shared across the clades. Although the allelic haplotypes were inferred based on short-read NGS using standard bioinformatic tools and have not been confirmed as physical haplotypes, they are the most likely existing combinations of the SNP sites. In fact, several of these inferred haplotypes were identical to the GenBank reference genome sequences, and some of those are shared between species. For example, the inferred haplotype 2 of *TLR7* we identified in *Equus hartmannae* and *Equus asinus* was identical to *Equus asinus* XP_014724205.1; similarly, the *TLR6* inferred haplotype 2 found in *Equus grevyi* was identical to *Equus quagga* XP_046512886.1. Nevertheless, all inferred haplotypes remain to be confirmed by long-read NGS.

Trans-species allele sharing due to polymorphism preceding speciation has been documented in immunity-related genes as well as in several other genes in humans and other species (Klumplerova et al. 2020; Azevedo et al. 2015; Halldórsdóttir and Árnason 2015). We observed trans-species allele sharing within the Equidae, while based on the few tapir and rhino sequences available, no alleles common to equids, tapirs and rhinoceroses, which diverged approximately 56 MY ago (Bai et al. 2018), were identified. Equids diverged into horses, zebras and asses approximately 4–5 MY ago (Librado and Orlando 2021). The existence of alleles shared across these clades after such a period of time may be explained by their adaptive value for the entire family, but also could be due to rapid speciation under strong negative selection. As a result, the branches of the constructed trees would not be well separated.

The two above explanations are not mutually exclusive. The adaptive value of *TLR* gene polymorphisms may also be estimated based on selection analyses of the sequences retrieved. Different types of selective pressures exerted on innate immunity genes may be reflected at the level of whole genes and/or at the level of selected amino acid sites. Liu et al. (2020) found evidence of purifying selection acting on entire vertebrate *TLR* genes, along with signatures of diversifying selection in specific codons. Here, we report that the same occurs in the Perissodactyla. All twelve TLR genes were under overall negative selection in perissodactyls. Significant negative selection was observed for *TLR* genes *3, 4, 7, 8, 9*, and *11* even in the relatively small group of equids. On the other hand, signatures of diversifying, site-specific selection were detected in each of the *TLR* genes in the group of perissodactyls as a whole (Fig. 3 and Online Resource 9). Some of these PSS remained significant when assessed only within the Equidae. This was the case for *TLR3, 10, 11, 12*. Some PSS were not located in the LRR domains as is usually observed (Downing et al. 2010; Velová et al. 2018), but were instead in the transmembrane region (TLR1) and in the cytoplasmic TIR domains (TLR9-12), which are typically conserved regions (Xu et al. 2000). Although PSS outside the LRR domains are rare, they have been reported (Areal et al. 2011). Notably, PSS E704G in TLR10 located in the TIR domain has the potential to affect protein function (SIFT score < 0.01) if glutamic acid is substituted by glycine. With a single exception, all other PSS with significant SIFT scores were located in the LRR or LRR-Ctd domains. Considering the role of the variability in LRR domains in the recognition of various PAMPs, this is not a surprising finding. A comparison of PSS rates with the variability of the overall amino acid sequence within each TLR showed no clear relationships between greater variation in the sequence and the number of amino acid sites under diversifying selection.

Twelve PSS that we identified in perissodactyls could be matched to PSS previously identified across vertebrates (Liu et al. 2020) and mammals (Areal et al. 2011). According to the SIFT analysis, amino acid variations at these sites did not have direct influence on the function of the protein. Nevertheless, the TLR4-A395 PSS, identified both by us as well as by both of the aforementioned studies, is located in the LRR domain and shows extensive variation (alanine, serine, threonine and arginine were all detected in perissodactyls), which may be related to the variability of the PAMPs recognized. In contrast to the two prior studies, we did not detect any signs of episodic diversifying selection in any of the TLR8 codons.

Based on their ligands and cell localization, two major subgroups of TLRs may be recognized. Receptors expressed on the cell membrane recognize primarily bacterial components (TLR 1,2,4,5,6). Receptors expressed on intracellular membranes (endosomes) (TLR 3,7,8,9) recognize viral nucleic acids (Kawai and Akira 2011). For the purpose of this analysis, we have expanded the non-viral group to include also TLR11 and TLR12, as they bind bacterial and protozoan motifs. Since TLR10 molecules recognize both bacterial and viral motifs and are mostly involved in anti-inflammatory responses (Su et al. 2021; Oosting et al. 2014), TLR10 was not included in this analysis. Differences in the selection patterns of viral and non-viral TLRs have been reported in primates (Barreiro et al. 2009) and carnivores (Liu et al. 2017), and these observations were extended to vertebrates by Liu et al. (2020). The authors showed that diversifying selection acted more strongly on non-viral TLRs, while viral TLRs were under stronger evolutionary constraints. A possible explanation is the higher redundancy and therefore evolutionary flexibility of non-viral TLRs, as bacteria display each several different PAMPs, which are detected simultaneously by different non-viral TLRs. In contrast, non-redundant intracellular viral sensors have only a narrow choice of targets (viral nucleic acids) and changes are not tolerated easily (Barreiro et al. 2009). In our results, diversifying selection prevailed in the non-viral group in perissodactyls, which is consistent with findings by Liu et al. (2020) for all vertebrates.

The adaptive value of Toll-like receptor polymorphisms may also be reflected in their associations with the host’s susceptibility or resistance to infectious diseases (Mukherjee et al. 2019). Based on the overall sequence similarities of *TLR* gene sequences across mammalian species, it is possible to compare amino acid sites associated with disease in one species with the selection status of those sites in another species. In our study, a PSS identified in TLR1 in perissodactyls (Y309Q, H) corresponded to the H305L site associated with pulmonary tuberculosis in humans (Ma et al. 2007; Meyer et al. 2016). I602S polymorphism is associated with aspergillosis (Kesh et al. 2005) and leprosy (Johnson et al. 2007) in humans; in equids, this site was also variable (I606V), but it was not a PSS. For TLR2, a F227L amino-acid change was associated with *Mycobacterium avium spp. paratuberculosis* infection in cattle (Fisher et al. 2011). A corresponding variable site F227L was also found in equids (but not in tapirs or rhinos) but with no signs of diversifying selection. In humans and mice, genetic polymorphisms in *TLR4* are involved in the LPS-induced signal transduction, but this was not confirmed for horses (Werners et al. 2006). The equine TLR4 site 420, participating in TLR4/MD2 binding, and sites 345, 364, 365, 367, 369, 385 and 414 that contribute to TLR4/TLR4 contact (Walsh et al. 2008), were variable in tapirs and rhinos, but not in equids. None of these sites were under positive selection. These comparisons show that amino acid sites of potential functional importance are often variable in equids or in perissodactyls. However, the significance of these variations in these species remains unknown.

## Conclusions

Toll-like receptors are core components of innate immunity. While most studies on TLRs are focused on humans and mice, only limited data are available for non-model species. This study identified twelve *TLR* genes in perissodactyls both by bioinformatic analyses and re-sequencing. The expression of *TLR11* and *TLR12* was confirmed by cDNA sequencing. Phylogenic reconstruction of the *TLR* gene family identified six sub-families. *TLR4* clustered together with *TLR5*; the *TLR1-6-10* subfamily showed a high degree of sequence identity corresponding to its evolution through gene conversion. In general terms, *TLR* genes are rather conserved in the family Equidae, similarly to other immunity. However, distinction between zebras and asses was not always observed, which may be due to selective pressures acting on these genes. Although phylogenetic trees constructed for each TLR did not show any major deviations from neutrality, trans-species sharing of inferred haplotypes was identified across all equids, but not across other perissodactyls. All twelve *TLR* genes were under strong negative overall selection. Signatures of diversifying selection in specific codon sites were detected in all TLRs except TLR8. Differences in the selection patterns of viral and non-viral TLRs were observed.

### Electronic supplementary material

Below is the link to the electronic supplementary material.

Supplementary Material 1

Supplementary Material 2

Supplementary Material 3

Supplementary Material 4

Supplementary Material 5

Supplementary Material 6

Supplementary Material 7

Supplementary Material 8

Supplementary Material 9

## Data Availability

All data generated or analysed during this study are included in this published article, its supplementary information files and/or the GenBank repository.
