# Supplementary material for: Twelve toll-like receptor (TLR) genes in the family Equidae – comparative genomics, selection and evolution
Source: Vet Res Commun. 2023 Oct 24;48(2):725–41. doi: 10.1007/s11259-023-10245-4 (PMC10998774; doi:10.1007/s11259-023-10245-4)
Supplement: Supplementary file 1 — Supplementary Material 1 [file 11259_2023_10245_MOESM1_ESM.docx]

The Toll-like receptor (TLR) genes in the family Equidae

Veterinary Research and Communications

Stejskalova 1 Department of Animal Genetics, Faculty of Veterinary Medicine, University of Veterinary Sciences Brno, 61242 Brno, Czech Republic

2 CEITEC VETUNI, RG Animal Immunogenomics, University of Veterinary Sciences Brno, Brno, Czech Republic

3 Zoo Prague, Prague, Czech Republic

* corresponding author: horin@ics.muni.cz

K.1, Janova E.1,2, Splichalova P.1, Futas J.1,2, Oppelt J.2, Vodicka R.3, Horin P.1,2,*

**Primers and protocols used for PCR amplification for NGS sequencing**

|  | forward | reverse | product lenght (bp) |  |  |  |
| --- | --- | --- | --- | --- | --- | --- |
| TLR1 | GTCGTGGGGGTATGTGTCAA | AACGGGGGCCTGAAAGTACA | 3544 |  |  |  |
| TLR2 | AGGGTGAAGTGCAGATTTGG | AGGGATATGGCCAGGCTTCTT | 3442 |  |  |  |
| TLR3a | AGATACTGGTTGCTTCCTAGCC | CAGCTCCTAATTGTCACCCAAC | 4084 |  |  |  |
| TLR3b | GACAGTGTATCCTGCCGTGTAA | GGAACACCTGAAGTAGGGAGAC | 3420 |  |  |  |
| TLR4a* | ACGGTGCGTCATGCTTTCAC | AACGAAGATGAGACTCCACCAA | 4358 |  |  |  |
| TLR4b* | CCTCTGCTTAGCATGTGTGC | TCATGACGGCCGTAACTTAG | 3363 |  |  |  |
| TLR5 | ATGTGAAGCAAAAATGTGTCTCTT | AGTGAAGAGTTTTCTTGGCTTGAG | 2996 |  |  |  |
| TLR6 | GTTTGAGAGCAATCATCCAGTCTT | GTGCAGATAATGGAGGCACAAT | 2620 |  |  |  |
| TLR7a | GCCTGGAGGTTCTGTGACTC | TCTCTCTTGCCAGCTGGTTT | 602 |  |  |  |
| TLR7b | GGTGGGGGTTGCTGTTTAG | CCAGTTACGATATCTGGACCTCA | 3846 |  |  |  |
| TLR8 | AGCACTGTGAAGCTGAACACA | GCACCTTGATCACAGCCTTT | 3530 |  |  |  |
| TLR9 | GACTTCAGACTGGGCTCTGG | GACCGCATTCAGTGACCTTC | 4750 |  |  |  |
| TLR10 | GAGAGGG GAATATGGCA CAG | AAACCTTTGCAAACTTGTGTTTC | 2883 |  |  |  |
| TLR11 | CTACTCGGCCAAATGTCTCCTC | TTTGACTTTTTGGACCTCCGGT | 3410 |  |  |  |
| TLR12 | TGAGGAGAAACCCCATTCTGAC | GAAGATGAGTTTGGTCTCCCGA | 3354 |  |  |  |

*primers and protocols for Sanger sequencing available upon request.

|  | mix | *ul* | cycling conditions |
| --- | --- | --- | --- |
|  |  |  |  |
|  | 2x PCR BIO Ultra Mix | 6,25 | 95°2m |
| **TLR1** | 10uM primers á | 0,5 | 95°15s |
|  | H2O | 4,25 | 62°15s |
|  | DNA | 1 | 72°1m, 35x |
|  |  |  |  |
|  | EliZyme HS Robust MIX Red | 6,25 | 95°3m |
| **TLR2** | 10uM primers á | 0,5 | 95°20s |
|  | H2O | 4,25 | 65°15s |
|  | DNA | 1 | 72°1m, 35x |
|  |  |  |  |
|  | 2x PCR BIO Ultra Mix | 6,25 | 95°2m |
| **TLR3a** | 10uM primers á | 0,5 | 95°15s |
|  | H2O | 4,25 | 66°15s |
|  | DNA | 1 | 72°1m, 32x |
|  |  |  |  |
|  |  |  |  |
|  | EliZyme HS Robust MIX Red | 6,25 | 95°2m |
| **TLR3b** | 10uM primers á | 0,5 | 95°15s |
|  | H2O | 4,25 | 60°15s |
|  | DNA | 1 | 72°50s, 35x |
|  |  |  |  |
|  | EliZyme HS Robust MIX Red | 6,25 | 95°3m |
|  | 10uM primers á | 0,5 | 95°30s |
| **TLR4b** | H2O | 4,25 | 64°20s |
|  | DNA | 1 | 72°1m30s, 34x |
|  |  |  |  |
|  |  |  | 92°2m |
|  | H2O | *7,2* | 92°10s |
|  | buffer with Mg | *2,5* | 65°15s |
| **TLR4a** | dNTPs | *0,625* | 68°4m, 10x |
|  | 10uM primers á | *0,5* | 92°10s |
|  | Roche ExpandLR | *0,175* | 65°15s |
|  | DNA | *1* | 68°4m+20s/cycle, 22x |
|  |  |  |  |
|  | 2x PCR BIO Ultra Mix | *6,25* | 95°2m |
| **TLR5** | 10uM primers á | *0,5* | 95°15s |
|  | H2O | *4,25* | 62°15s |
|  | DNA | *1* | 72°50s, 35x |
|  |  |  |  |
|  | EliZyme HS Robust MIX Red RED | 6,25 | 95°3m |
| **TLR6** | 10uM primers á | 0,5 | 95°30s |
|  | H2O | 4,25 | 62°15s |
|  | DNA | 1 | 72°1m, 35x |
|  |  |  |  |
|  | EliZyme HS Robust MIX Red RED | 6,25 | 95°2m |
| **TLR7a** | 10uM primers á | 0,5 | 95°15s |
|  | H2O | 4,25 | 60°15s |
|  | DNA | 1 | 72°10s, 39x |
|  |  |  |  |
|  | EliZyme HS Robust MIX Red RED | 6,25 | 95°2m |
| **TLR7b** | 10uM primers á | 0,5 | 95°20s |
|  | H2O | 4,25 | 64°15s |
|  | DNA | 1 | 72°1min, 35x |
|  |  |  |  |
|  | EliZyme HS Robust MIX Red RED | 6,25 | 95°2m |
| **TLR8** | 10uM primers á | 0,5 | 95°20s |
|  | H2O | 4,25 | 64°15s |
|  | DNA | 1 | 72°50s, 35x |
|  |  |  |  |
|  |  |  | 92°2m |
|  | H2O | *7,2* | 92°10s |
|  | buffer with Mg | *2,5* | 62°15s |
| **TLR9** | dNTPs | *0,625* | 68°5m, 10x |
|  | 10uM primers á | *0,5* | 92°10s |
|  | Roche ExpandLR | *0,175* | 62°15s |
|  | DNA | *1* | 68°5m+20s/cycle, 22x |
|  |  |  |  |
|  | EliZyme HS Robust MIX Red RED | 6,25 | 95°2m |
| **TLR10** | 10uM primers á | 0,5 | 95°20s |
|  | H2O | 4,25 | 63°15s |
|  | DNA | 1 | 72°50s, 37x |
|  |  |  |  |
|  | 2x PCR BIO Ultra Mix | *6,25* | 95°2m |
| **TLR11** | 10uM primers á | *0,5* | 95°15s |
|  | H2O | *4,25* | 64°15s |
|  | DNA | *1* | 72°50s, 35x |
|  |  |  |  |
|  | 2x PCR BIO Ultra Mix | *6,25* | 95°2m |
| **TLR12** | 10uM primers á | *0,5* | 95°15s |
|  | H2O | *4,25* | 62°15s |
|  | DNA | *1* | 72°50s, 35x |

**Primers used for PCR amplification for cDNA amplification**

|  | forward | reverse | product lenght (bp) |  |  |  |
| --- | --- | --- | --- | --- | --- | --- |
| TLR11 | AGTCGTGAGAACTGAAATCCCC | TTTGACTTTTTGGACCTCCGGT | 3605 |  |  |  |
|  |  |  |  |  |  |  |
| TLR12 | CCGTCGGTGCTACTAGGCTT | GAAGATGAGTTTGGTCTCCCGA | 2856 |  |  |  |

|  | HotStarTaq Plus MasterMix | *6,25* | 95°5m |
| --- | --- | --- | --- |
| **TLR11** | primers á | *0,5* | 95°1m |
|  | H2O | *4,25* | 60°30s |
|  | cDNA | *1* | 72°3m, 35x |
|  |  |  |  |
|  | HotStarTaq Plus MasterMix | *6,25* | 95°5m |
| **TLR12** | primers á | *0,5* | 95°1m |
|  | H2O | *4,25* | 60°30s |
|  | cDNA | *1* | 72°3m, 35x |
