# Supplementary material for: Twelve toll-like receptor (TLR) genes in the family Equidae – comparative genomics, selection and evolution
Source: Vet Res Commun. 2023 Oct 24;48(2):725–41. doi: 10.1007/s11259-023-10245-4 (PMC10998774; doi:10.1007/s11259-023-10245-4)
Supplement: Supplementary file 2 — Supplementary Material 2 [file 11259_2023_10245_MOESM2_ESM.docx]

The Toll-like receptor (TLR) genes in the family Equidae

Veterinary Research and Communications

Stejskalova K.1, Janova E.1,2, Splichalova P.1, Futas J.1,2, Oppelt J.2, Vodicka R.3, Horin P.1,2,*

1 Department of Animal Genetics, Faculty of Veterinary Medicine, University of Veterinary Sciences Brno, 61242 Brno, Czech Republic

2 CEITEC VETUNI, RG Animal Immunogenomics, University of Veterinary Sciences Brno, Brno, Czech Republic

3 Zoo Prague, Prague, Czech Republic

* corresponding author: horin@ics.muni.cz

GenBank online resources for Perissodactyla, Homo sapiens, Mus musculus and Bos taurus:

TLR1

| TaIn TLR1 PVIE01002705.1:28853-31210 |
| --- |
| TaTe TLR1 PVID01005798.1:59099-61456 |
| RhUn TLR1 BOSQ02005794.1:477295-479661 |
| DiBi TLR1 CM036953.1:31922719-31925085 |
| DiSu TLR1 PEKH010002443.1:1125503-1127869 |
| CeSi TLR1 XP_004419033.1 |
| HoSa TLR1 NP_003254.2 |
| BoTa TLR1 FJ147090.1 |
| MuMu TLR1 NM_001276445.1 |

TLR2

| TaIn TLR2 PVIE01002162.1:295166-297520 |
| --- |
| DiBi TLR2 CM036956.1:41860469-41862820 |
| DiSu TLR2 PEKH010005480.1:508458-510809 |
| CeSi TLR2 XP_004420987.1 |
| BoTa TLR2 NP_776622.1 |
| MuMu TLR2 NM_011905.3 |
| HoSa TLR2 NM_001318787.2 |
| TLR3 |
| \| TaIn TLR3 PVIE01002130.1 merged blast hits \| \| --- \| \| TaTe TLR3 PVID01001904.1 merged blast hits \| \| CeSi TLR3 XP_004428823.1 \| \| RhUn TLR3 BOSQ02001280.1 merged blast hits \| \| Disu TLR3 PEKH011061517.1 merged blast hits \| \| DiBi TLR3 CM036974.1 merged blast hits \| \| BoTa TLR3 NM_001008664.1 \| \| MuMu TLR3 NM_126166.5_ \| \| HoSa TLR3 NP_003256.1 \|   TLR4 |
| \| TaIn TLR4 PVIE01000108.1 merged blast hits \| \| --- \| \| TaTe TLR4 PVID01005477.1 merged blast hits \| \| RhUn TLR4 BOSQ02004256.1 merged blast hits \| \| DiBi TLR4 CM036973.1 merged blast hits \| \| DiSu TLR4 PEKH010006957.1 merged blast hits \| \| CeSi TLR4 XP_004423427.1 \| \| BoTa TLR4 NP_776623.5 \| \| HoSa TLR4_NP_612564.1_ \| \| MuMu TLR4 NM_021297.3 \|   TLR5 |
| \| TaIn TLR5 PVIE01002717.1:193380-195956 \| \| --- \| \| TaTe TLR5 PVID01000437.1:270451-273027 \| \| RhUn TLR5 BOSQ02004549.1:253115-255688 \| \| DiBi TLR5 CM036983.1:10323539-10326112 \| \| DiSu TLR5 PEKH010003866.1:1389063-1391636 \| \| CeSi TLR5 XP_004439593.1 \| \| BoTa TLR5 NP_001035591.1 \| \| HoSa TLR5 NM_003268.6_ \|   TLR6   \| TaIn TLR6 PVIE01002705.1:55589-57976 \| \| --- \| \| TaTe TLR6 PVID01005798.1:86077-88464 \| \| RhUn TLR6 BOSQ02005794.1:504727-507112 \| \| DiBi TLR6 CM036953.1:31950039-31952423 \| \| DiSu TLR6 PEKH010002443.1:1152593-1154977 \| \| CeSi TLR6 XP_004419035.1 \| \| BoTa TLR6 NP_001001159.1 \| \| MuMu TLR6 NM_011604.5 \| \| HoSa TLR6 NM_006068.5 \| |
| TLR7   \| TaIn TLR7 PVIE01001303.1 merged blast hits \| \| --- \| \| TaTe TLR7 PVID01005005.1 merged blast hits \| \| RhUn TLR7 BOSQ02004008.1 merged blast hits \| \| DiBi TLR7 CM036987.1 merged blast hits \| \| DiSu TLR7 PEKH010009301.1 merged blast hits \| \| CeSi TLR7 XP_004435171.1 \| \| BoTa TLR7 NP_001028933.1 \| \| HoSa TLR7 NM_016562.4_ \| \| MuMu TLR7 NM_001290755.1 \| |
| TLR8   \| TaIn TLR8 PVIE01001303.1:120434-123547 \| \| --- \| \| TaTe TLR8 PVID01005005.1:114309-117422 \| \| RhUn TLR8 BOSQ02006735.1:443-3556 \| \| DiBi TLR8 CM036987.1:10352427-10355540 \| \| DiSu TLR8 PEKH010009301.1:384065-387178 \| \| CeSi TLR8 XP_004435172.1 \| \| BoTa TLR8 NP_001029109.1 \| \| HoSa TLR8 NP_057694.2_ \| \| MuMu TLR8 NM_133212.3_ \| |
| TLR9   \| TaIn TLR9 PVIE01001527.1:57233-57235 56373- 53281 \| \| --- \| \| TaTe TLR9 PVID01000760.1:338387-341480 \| \| RhUn TLR9 BOSQ02002635.1:255318-258408 \| \| DiBi TLR9 CM036947.1:52885164-52888254 \| \| DiSu TLR9 PEKH010003248.1:336582-339672 \| \| CeSi TLR9 XP_004419841.1 \| \| BoTa TLR9 NP_898904.1 \| \| HoSa TLR9_NP_059138.1 \| \| MuMu TLR9 NM_031178.2_ \| |
| TLR10   \| TaIn TLR10 PVIE01002705.1:10600-13135 \| \| --- \| \| Tate TLR10 PVID01005798.1:40338-42773 \| \| RhUn TLR10 BOSQ02005794.1: 454423-456855 \| \| DiBi TLR10CM036953.1:31900945-31903377 \| \| DiSu TLR10 PEKH010002443.1:1104227-1106654 \| \| CeSi TLR10 XP_014642233.1 \| \| BoTa TLR10 NP_001070386.1 \| \| HoSa TLR10 NP_001182035.1 \| |
| TLR11   \| TaIn TLR11 PVIE01009921.1:4996-7764 \| \| --- \| \| TaTe TLR11 PVID01008473.1:27101-29869 \| \| DiBi TLR11 CM036950.1:29505321-29508089 \| \| DiSu TLR11 PEKH010006267.1:500232-503000 \| \| CeSi TLR11 XP_004421380.2 \| \| MuMu TLR11 NP_991388.2 \| |
| TLR12   \| TaIn TLR12 PVIE01004340.1:50200-52970 \| \| --- \| \| TaTe TLR12 PVID01000256.1:113166-115892 \| \| RhUn TLR12 BOSQ02002621.1:1435703-1438429 \| \| DiBi TLR12 CM036958.1:47967698-47970424 \| \| DiSu TLR12 PEKH010004974.1:381217-383943 \| \| CeSi TLR12 XP_004426196.1 \| \| MuMu TLR12 NP_991392.1 \| |
