# Supplementary material for: Twelve toll-like receptor (TLR) genes in the family Equidae – comparative genomics, selection and evolution
Source: Vet Res Commun. 2023 Oct 24;48(2):725–41. doi: 10.1007/s11259-023-10245-4 (PMC10998774; doi:10.1007/s11259-023-10245-4)
Supplement: Supplementary file 5 — Supplementary Material 5 [file 11259_2023_10245_MOESM5_ESM.docx]

The Toll-like receptor (TLR) genes in the family Equidae

Veterinary Research and Communications

Stejskalova K.1, Janova E.1,2, Splichalova P.1, Futas J.1,2, Oppelt J.2, Vodicka R.3, Horin P.1,2,*

1 Department of Animal Genetics, Faculty of Veterinary Medicine, University of Veterinary Sciences Brno, 61242 Brno, Czech Republic

2 CEITEC VETUNI, RG Animal Immunogenomics, University of Veterinary Sciences Brno, Brno, Czech Republic

3 Zoo Prague, Prague, Czech Republic

* corresponding author: horin@ics.muni.cz

# TLR1

| GenBank accession number | alignment seq name |
| --- | --- |
| OR021987 | EqGr_TLR1_heq1 |
| OR021988 | EqGr_TLR1_heq2 |
| OR021989 | EqQuAn_TLR1_heq4 |
| OR021990 | EqQuAn_TLR1_heq5 |
| OR021991 | EqQuBoe_TLR1_heq6 |
| OR021992 | EqQuBoe_TLR1_heq7 |
| OR021993 | EqQuBor_TLR1_heq10 |
| OR021994 | EqQuBor_TLR1_heq11 |
| OR021995 | EqQuCh_TLR1_heq8 |
| OR021996 | EqQuCh_TLR1_heq9 |
| OR021997 | EqHa_TLR1_heq3 |
| OR021998 | EqAs_TLR1_heq14 |
| OR021999 | EqAfSo_TLR1_heq13 |
| OR022000 | EqAfSo_TLR1_heq12 |
| OR022001 | EqHeKu_TLR1_heq18 |
| OR022002 | EqHeKu_TLR1_heq19 |
| OR022003 | EqKi_TLR1_heq15 |
| OR022004 | EqKi_TLR1_heq16 |
| OR022005 | EqKi_TLR1_heq17 |
| OR022006 | EqPr_TLR1_heq20 |
| OR022007 | EqPr_TLR1_heq22 |
| OR022008 | EqPr_TLR1_heq23 |
| OR022009 | EqCa_TLR1_heq20 |
| OR022010 | EqCa_TLR1_heq21 |
| OR022011 | EqCa_TLR1_heq22 |

# TLR2

| GenBank accession number | alignment seq name |
| --- | --- |
| OR061136 | EqGr_TLR2_Heq1 |
| OR061137 | EqGr_TLR2_Heq2 |
| OR061138 | EqQuAn_TLR2_Heq6 |
| OR061139 | EqQuAn_TLR2_Heq7 |
| OR061140 | EqQuBoe_TLR2_Heq8 |
| OR061141 | EqQuBoe_TLR2_Heq9 |
| OR061142 | EqQuBoe_TLR2_Heq10 |
| OR061143 | EqQuBor_TLR2_Heq10 |
| OR061144 | EqQuBor_TLR2_Heq13 |
| OR061145 | EqQuCh_TLR2_Heq11 |
| OR061146 | EqQuCh_TLR2_Heq12 |
| OR061147 | EqQuCh_TLR2_Heq6 |
| OR061148 | EqHa_TLR2_Heq3 |
| OR061149 | EqHa_TLR2_Heq4 |
| OR061150 | EqHa_TLR2_Heq5 |
| OR061151 | EqAs_TLR2_Heq16 |
| OR061152 | EqAs_TLR2_Heq17 |
| OR061153 | EqAfSo_TLR2_Heq15 |
| OR061154 | EqAfSo_TLR2_Heq14 |
| OR061155 | EqHeKu_TLR2_Heq20 |
| OR061156 | EqKi_TLR2_Heq18 |
| OR061157 | EqKi_TLR2_Heq19 |
| OR061158 | EqPr_TLR2_Heq21 |
| OR061159 | EqPr_TLR2_Heq22 |
| OR061160 | EqCa_TLR2_Heq23 |
| OR061161 | EqCa_TLR2_Heq21 |
| OR061162 | EqCa_TLR2_Heq24 |

# TLR3

| GenBank accession number | alignment seq name |
| --- | --- |
| OR077157 | EqGr_TLR3_heq1 |
| OR077158 | EqQuAn_TLR3_heq1 |
| OR077159 | EqQuBoe_TLR3_heq1 |
| OR077160 | EqQuBor_TLR3_heq1 |
| OR077161 | EqGr_TLR3_heq2 |
| OR077162 | EqHa_TLR3_heq3 |
| OR077163 | EqHa_TLR3_heq4 |
| OR077164 | EqQuBoe_TLR3_heq4 |
| OR077165 | EqQuCh_TLR3_heq4 |
| OR077166 | EqQuAn_TLR3_heq5 |
| OR077167 | EqQuAn_TLR3_heq6 |
| OR077168 | EqQuBoe_TLR3_heq7 |
| OR077169 | EqQuBoe_TLR3_heq8 |
| OR077170 | EqQuCh_TLR3_heq9 |
| OR077171 | EqQuCh_TLR3_heq10 |
| OR077172 | EqQuCh_TLR3_heq11 |
| OR077173 | EqQuBor_TLR3_heq12 |
| OR077174 | EqQuBor_TLR3_heq13 |
| OR077175 | EqAfSo_TLR3_heq14 |
| OR077176 | EqAs_TLR3_heq14 |
| OR077177 | EqAfSo_TLR3_heq15 |
| OR077178 | EqAfSo_TLR3_heq16 |
| OR077179 | EqAs_TLR3_heq17 |
| OR077180 | EqKi_TLR3_heq18 |
| OR077181 | EqKi_TLR3_heq19 |
| OR077182 | EqHeKu_TLR3_heq20 |
| OR077183 | EqHeKu_TLR3_heq21 |
| OR077184 | EqCa_TLR3_heq22 |
| OR077185 | EqPr_TLR3_heq22 |
| OR077186 | EqCa_TLR3_heq23 |
| OR077187 | EqPr_TLR3_heq24 |
| OR077188 | EqPr_TLR3_heq25 |
| OR077189 | EqPr_TLR3_heq26 |
| TLR4 GenBank accession number | alignment seq name |
| OR077190 | EqGr_TLR4_Heq1 |
| OR077191 | EqGr_TLR4_Heq2 |
| OR077192 | EqQuAn_TLR4_Heq4 |
| OR077193 | EqQuAn_TLR4_Heq5 |
| OR077194 | EqQuBoe_TLR4_Heq6 |
| OR077195 | EqQuBoe_TLR4_Heq7 |
| OR077196 | EqQuBor_TLR4_Heq8 |
| OR077197 | EqQuBor_TLR4_Heq6 |
| OR077198 | EqQuBor_TLR4_Heq9 |
| OR077199 | EqQuCh_TLR4_Heq6 |
| OR077200 | EqHa_TLR4_Heq3 |
| OR077201 | EqAs_TLR4_Heq10 |
| OR077202 | EqAs_TLR4_Heq12 |
| OR077203 | EqAfSo_TLR4_Heq10 |
| OR077204 | EqAfSo_TLR4_Heq11 |
| OR077205 | EqHeKu_TLR4_Heq14 |
| OR077206 | EqHeKu_TLR4_Heq13 |
| OR077207 | EqHeKu_TLR4_Heq15 |
| OR077208 | EqKi_TLR4_Heq13 |
| OR077209 | EqPr_TLR4_Heq19 |
| OR077210 | EqPr_TLR4_Heq20 |
| OR077211 | EqPr_TLR4_Heq16 |
| OR077212 | EqCa_TLR4_Heq16 |
| OR077213 | EqCa_TLR4_Heq17 |
| OR077214 | EqCa_TLR4_Heq18 |

# TLR5

| GenBank accession number | alignment seq name |
| --- | --- |
| OR061163 | EqGr_TLR5_Heq5 |
| OR061164 | EqGr_TLR5_Heq6 |
| OR061165 | EqQuAn_TLR5_Heq15 |
| OR061166 | EqQuAn_TLR5_Heq16 |
| OR061167 | EqQuBoe_TLR5_Heq17 |
| OR061168 | EqQuBoe_TLR5_Heq18 |
| OR061169 | EqQuBoe_TLR5_Heq19 |
| OR061170 | EqQuBor_TLR5_Heq20 |
| OR061171 | EqQuBor_TLR5_Heq15 |
| OR061172 | EqQuBor_TLR5_Heq21 |
| OR061173 | EqQuCh_TLR5_Heq15 |
| OR061174 | EqQuCh_TLR5_Heq22 |
| OR061175 | EqHa_TLR5_Heq23 |
| OR061176 | EqHa_TLR5_Heq24 |
| OR061177 | EqAs_TLR5_Heq1 |
| OR061178 | EqAfSo_TLR5_Heq2 |
| OR061179 | EqAfSo_TLR5_Heq3 |
| OR061180 | EqHeKu_TLR5_Heq7 |
| OR061181 | EqHeKu_TLR5_Heq8 |
| OR061182 | EqHeKu_TLR5_Heq9 |
| OR061183 | EqHeKu_TLR5_Heq10 |
| OR061184 | EqKi_TLR5_Heq11 |
| OR061185 | EqKi_TLR5_Heq12 |
| OR061186 | EqPr_TLR5_Heq13 |
| OR061187 | EqPr_TLR5_Heq14 |
| OR061188 | EqPr_TLR5_Heq1 |
| OR061189 | EqCa_TLR5_Heq4 |
| OR061190 | EqCa_TLR5_Heq1 |

# TLR6

| GenBank accession number | alignment seq name |
| --- | --- |
| OR061191 | EqGr_TLR6_Heq1 |
| OR061192 | EqGr_TLR6_Heq2 |
| OR061193 | EqQuAn_TLR6_Heq4 |
| OR061194 | EqQuAn_TLR6_Heq5 |
| OR061195 | EqQuAn_TLR6_Heq2 |
| OR061196 | EqQuBoe_TLR6_Heq2 |
| OR061197 | EqQuBoe_TLR6_Heq6 |
| OR061198 | EqQuBor_TLR6_Heq2 |
| OR061199 | EqQuCh_TLR6_Heq7 |
| OR061200 | EqQuCh_TLR6_Heq8 |
| OR061201 | EqQuCh_TLR6_Heq2 |
| OR061202 | EqHa_TLR6_Heq3 |
| OR061203 | EqAs_TLR6_Heq10 |
| OR061204 | EqAfSo_TLR6_Heq9 |
| OR061205 | EqHeKu_TLR6_Heq13 |
| OR061206 | EqHeKu_TLR6_Heq14 |
| OR061207 | EqKi_TLR6_Heq12 |
| OR061208 | EqKi_TLR6_Heq11 |
| OR061209 | EqPr_TLR6_Heq19 |
| OR061210 | EqPr_TLR6_Heq18 |
| OR061211 | EqPr_TLR6_Heq20 |
| OR061212 | EqPr_TLR6_Heq17 |
| OR061213 | EqPr_TLR6_Heq16 |
| OR061214 | EqCa_TLR6_Heq15 |
| OR061215 | EqCa_TLR6_Heq16 |
| OR061216 | EqCa_TLR6_Heq17 |

# TLR7

| GenBank accession number | alignment seq name |
| --- | --- |
| OR077215 | EqGr_TLR7_Heq1 |
| OR077216 | EqQuAn_TLR7_Heq3 |
| OR077217 | EqQuAn_TLR7_Heq4 |
| OR077218 | EqQuBoe_TLR7_Heq5 |
| OR077219 | EqQuBor_TLR7_Heq8 |
| OR077220 | EqQuBor_TLR7_Heq9 |
| OR077221 | EqQuCh_TLR7_Heq6 |
| OR077222 | EqQuCh_TLR7_Heq7 |
| OR077223 | EqHa_TLR7_Heq2 |
| OR077224 | EqAs_TLR7_Heq2 |
| OR077225 | EqAs_TLR7_Heq10 |
| OR077226 | EqAfSo_TLR7_Heq2 |
| OR077227 | EqHeKu_TLR7_Heq11 |
| OR077228 | EqKi_TLR7_Heq11 |
| OR077229 | EqKi_TLR7_Heq12 |
| OR077230 | EqKi_TLR7_Heq13 |
| OR077231 | EqPr_TLR7_Heq14 |
| OR077232 | EqPr_TLR7_Heq17 |
| OR077233 | EqCa_TLR7_Heq14 |
| OR077234 | EqCa_TLR7_Heq15 |
| OR077235 | EqCa_TLR7_Heq16 |

# TLR8

| GenBank accession number | alignment seq name |
| --- | --- |
| OR061217 | EqGr_TLR8_Heq1 |
| OR061218 | EqQuAn_TLR8_Heq4 |
| OR061219 | EqQuAn_TLR8_Heq5 |
| OR061220 | EqQuBoe_TLR8_Heq6 |
| OR061221 | EqQuBor_TLR8_Heq4 |
| OR061222 | EqQuBor_TLR8_Heq6 |
| OR061223 | EqQuCh_TLR8_Heq6 |
| OR061224 | EqQuCh_TLR8_Heq4 |
| OR061225 | EqHa_TLR8_Heq2 |
| OR061226 | EqHa_TLR8_Heq3 |
| OR061227 | EqAs_TLR8_Heq7 |
| OR061228 | EqAfSo_TLR8_Heq7 |
| OR061229 | EqAfSo_TLR8_Heq8 |
| OR061230 | EqHeKu_TLR8_Heq11 |
| OR061231 | EqKi_TLR8_Heq2 |
| OR061232 | EqKi_TLR8_Heq9 |
| OR061233 | EqKi_TLR8_Heq10 |
| OR061234 | EqPr_TLR8_Heq15 |
| OR061235 | EqPr_TLR8_Heq13 |
| OR061236 | EqPr_TLR8_Heq14 |
| OR061237 | EqCa_TLR8_Heq12 |
| OR061238 | EqCa_TLR8_Heq13 |
| OR061239 | EqCa_TLR8_Heq14 |

# TLR9

| GenBank accession number | alignment seq name |
| --- | --- |
| OR077236 | EqGr_TLR9_Heq1 |
| OR077237 | EqGr_TLR9_Heq2 |
| OR077238 | EqQuAn_TLR9_Heq5 |
| OR077239 | EqQuAn_TLR9_Heq6 |
| OR077240 | EqQuAn_TLR9_Heq2 |
| OR077241 | EqQuAn_TLR9_Heq7 |
| OR077242 | EqQuBoe_TLR9_Heq5 |
| OR077243 | EqQuBor_TLR9_Heq11 |
| OR077244 | EqQuBor_TLR9_Heq8 |
| OR077245 | EqQuBor_TLR9_Heq12 |
| OR077246 | EqQuCh_TLR9_Heq8 |
| OR077247 | EqQuCh_TLR9_Heq9 |
| OR077248 | EqQuCh_TLR9_Heq10 |
| OR077249 | EqHa_TLR9_Heq3 |
| OR077250 | EqHa_TLR9_Heq4 |
| OR077251 | EqAs_TLR9_Heq13 |
| OR077252 | EqAs_TLR9_Heq14 |
| OR077253 | EqAfSo_TLR9_Heq13 |
| OR077254 | EqAfSo_TLR9_Heq14 |
| OR077255 | EqHeKu_TLR9_Heq18 |
| OR077256 | EqHeKu_TLR9_Heq17 |
| OR077257 | EqKi_TLR9_Heq16 |
| OR077258 | EqKi_TLR9_Heq15 |
| OR077259 | EqPr_TLR9_Heq19 |
| OR077260 | EqPr_TLR9_Heq20 |
| OR077261 | EqPr_TLR9_Heq21 |
| OR077262 | EqPr_TLR9_Heq22 |
| OR077263 | EqCa_TLR9_Heq19 |
| OR077264 | EqCa_TLR9_Heq23 |
| OR077265 | EqCa_TLR9_Heq21 |

# TLR10

| GenBank accession number | alignment seq name |
| --- | --- |
| OR061240 | EqGr_TLR10_Heq1 |
| OR061241 | EqGr_TLR10_Heq2 |
| OR061242 | EqQuAn_TLR10_Heq2 |
| OR061243 | EqQuAn_TLR10_Heq4 |
| OR061244 | EqQuBoe_TLR10_Heq2 |
| OR061245 | EqQuBoe_TLR10_Heq5 |
| OR061246 | EqQuBor_TLR10_Heq2 |
| OR061247 | EqQuBor_TLR10_Heq6 |
| OR061248 | EqQuCh_TLR10_Heq2 |
| OR061249 | EqHa_TLR10_Heq3 |
| OR061250 | EqAs_TLR10_Heq8 |
| OR061251 | EqAs_TLR10_Heq7 |
| OR061252 | EqAs_TLR10_Heq9 |
| OR061253 | EqAfSo_TLR10_Heq7 |
| OR061254 | EqHeKu_TLR10_Heq12 |
| OR061255 | EqKi_TLR10_Heq10 |
| OR061256 | EqKi_TLR10_Heq11 |
| OR061257 | EqPr_TLR10_Heq13 |
| OR061258 | EqPr_TLR10_Heq16 |
| OR061259 | EqPr_TLR10_Heq14 |
| OR061260 | EqCa_TLR10_Heq13 |
| OR061261 | EqCa_TLR10_Heq14 |
| OR061262 | EqCa_TLR10_Heq15 |

# TLR11

| GenBank accession number | alignment seq name |
| --- | --- |
| OR061263 | EqGr_TLR11_Heq19 |
| OR061264 | EqGr_TLR11_Heq20 |
| OR061265 | EqGr_TLR11_Heq15 |
| OR061266 | EqQuAn_TLR11_Heq9 |
| OR061267 | EqQuAn_TLR11_Heq12 |
| OR061268 | EqQuAn_TLR11_Heq13 |
| OR061269 | EqQuBoe_TLR11_Heq13 |
| OR061270 | EqQuBor_TLR11_Heq13 |
| OR061271 | EqQuCh_TLR11_Heq8 |
| OR061272 | EqQuCh_TLR11_Heq9 |
| OR061273 | EqHa_TLR11_Heq10 |
| OR061274 | EqHa_TLR11_Heq11 |
| OR061275 | EqAs_TLR11_Heq1 |
| OR061276 | EqAs_TLR11_Heq2 |
| OR061277 | EqAs_TLR11_Heq4 |
| OR061278 | EqAfSo_TLR11_Heq5 |
| OR061279 | EqAfSo_TLR11_Heq6 |
| OR061280 | EqAfSo_TLR11_Heq7 |
| OR061281 | EqHeKu_TLR11_Heq18 |
| OR061282 | EqKi_TLR11_Heq14 |
| OR061283 | EqPr_TLR11_Heq16 |
| OR061284 | EqPr_TLR11_Heq17 |
| OR061285 | EqCa_TLR11_Heq1 |
| OR061286 | EqCa_TLR11_Heq2 |
| OR061287 | EqCa_TLR11_Heq3 |

# TLR12

| GenBank accession number | alignment seq name |
| --- | --- |
| OR061288 | EqGr TLR12 heq6 |
| OR061289 | EqGr TLR12 heq7 |
| OR061290 | EqGr TLR12 heq8 |
| OR061291 | EqQuAn TLR12 heq10 |
| OR061292 | EqQuAn TLR12 heq11 |
| OR061293 | EqQuBoe TLR12 heq20 |
| OR061294 | EqQuBoe TLR12 heq21 |
| OR061295 | EqQuBoe TLR12 heq22 |
| OR061296 | EqQuBor TLR12 heq22 |
| OR061297 | EqQuBor TLR12 heq25 |
| OR061298 | EqQuBor TLR12 heq21 |
| OR061299 | EqQuBor TLR12 heq26 |
| OR061300 | EqQuCh TLR12 heq13 |
| OR061301 | EqQuCh TLR12 heq14 |
| OR061302 | EqQuCh TLR12 heq10 |
| OR061303 | EqQuCh TLR12 heq18 |
| OR061304 | EqHa TLR12 heq12 |
| OR061305 | EqHa TLR12 heq17 |
| OR061306 | EqAs TLR12 heq27 |
| OR061307 | EqAs TLR12 heq3 |
| OR061308 | EqAfSo TLR12 heq9 |
| OR061309 | EqCa TLR12 heq24 |
| OR061310 | EqHeKu TLR12 heq19 |
| OR061311 | EqHeKu TLR12 heq23 |
| OR061312 | EqKi TLR12 heq5 |
| OR061313 | EqKi TLR12 heq15 |
| OR061314 | EqKi TLR12 heq16 |
| OR061315 | EqPr TLR12 heq3 |
| OR061316 | EqPr TLR12 heq4 |
| OR061317 | EqPr TLR12 heq1 |
| OR061318 | EqPr TLR12 heq2 |
| OR061319 | EqCa TLR12 heq3 |
| OR061320 | EqCa TLR12 heq1 |
