# Supplementary figures and images for: Twelve toll-like receptor (TLR) genes in the family Equidae – comparative genomics, selection and evolution
Source: Vet Res Commun. 2023 Oct 24;48(2):725–41. doi: 10.1007/s11259-023-10245-4 (PMC10998774; doi:10.1007/s11259-023-10245-4)

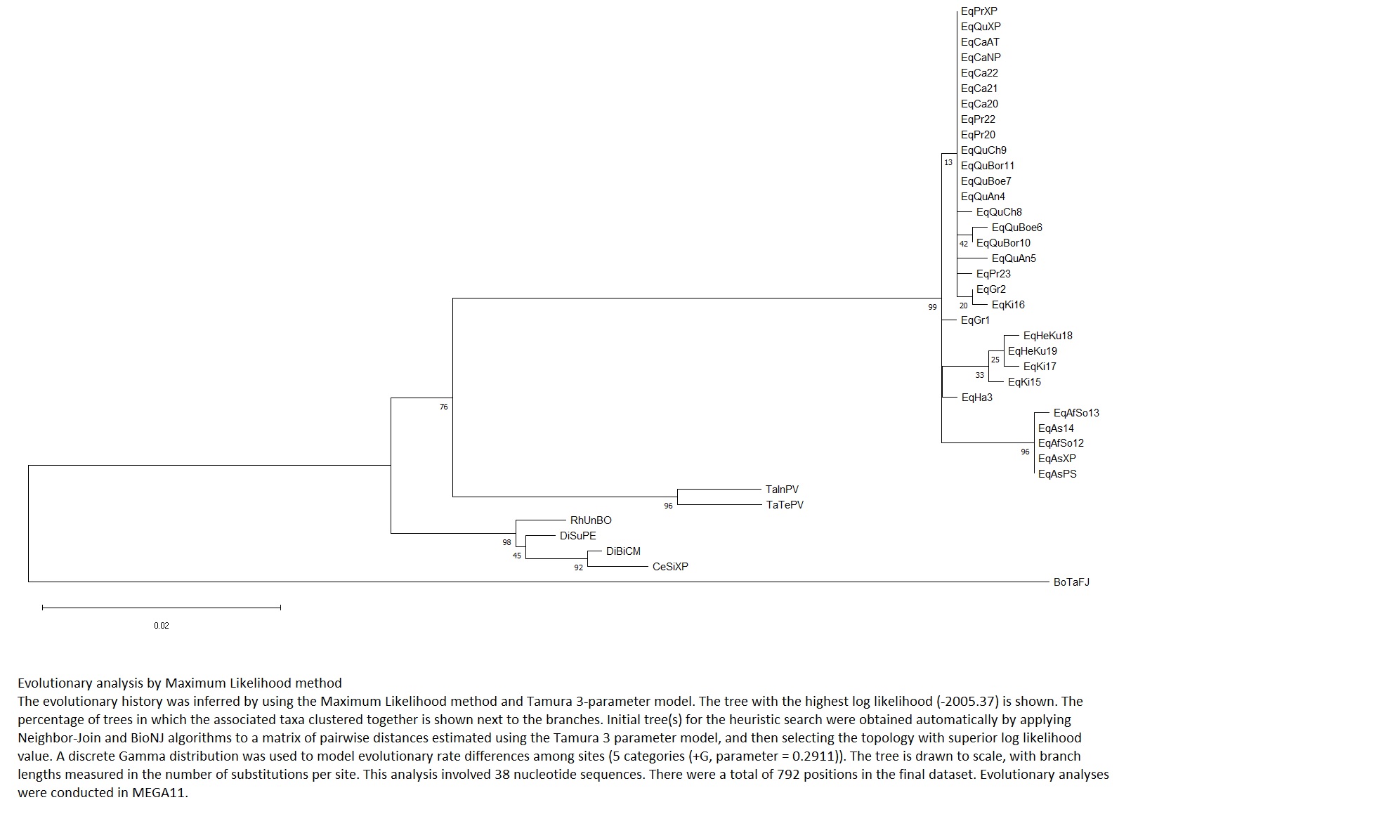

Supplement: Supplementary file 8 — Supplementary Material 8 [file 11259_2023_10245_MOESM8_ESM.zip › TLR1 ML perissodactyls.jpg]

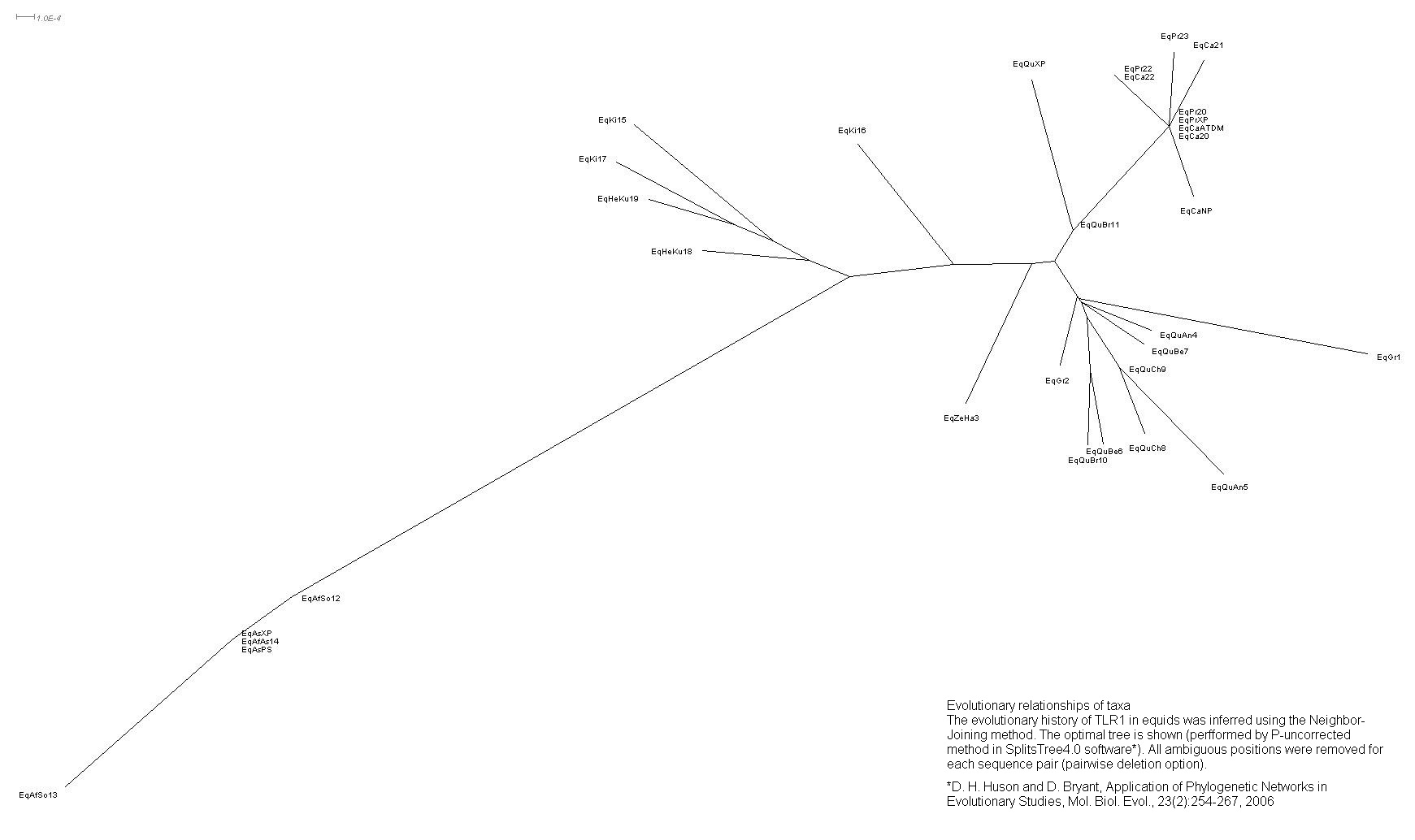

Supplement: Supplementary file 8 — Supplementary Material 8 [file 11259_2023_10245_MOESM8_ESM.zip › TLR1 NJtree equids.jpg]

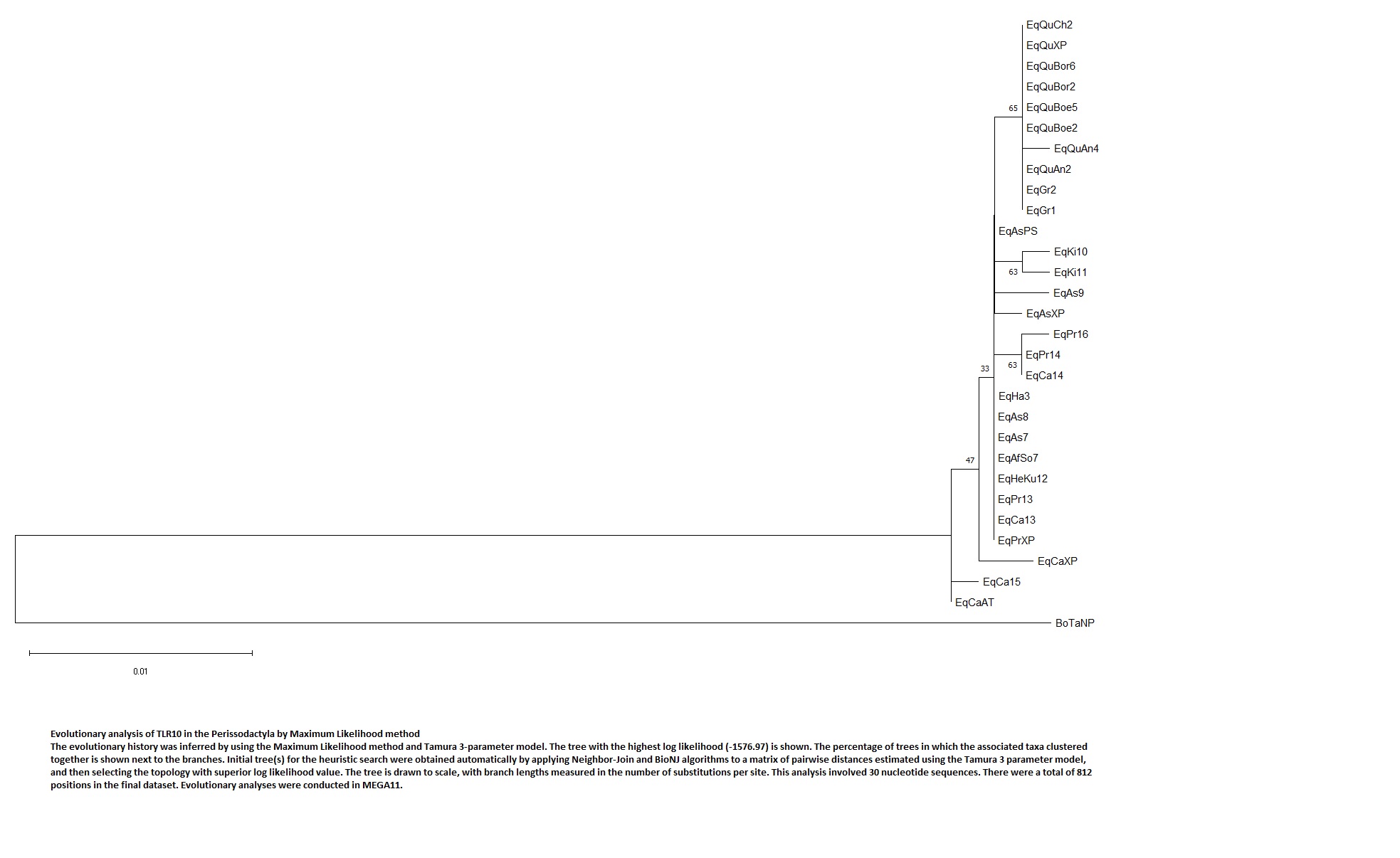

Supplement: Supplementary file 8 — Supplementary Material 8 [file 11259_2023_10245_MOESM8_ESM.zip › TLR10 ML perissodactyls.jpg]

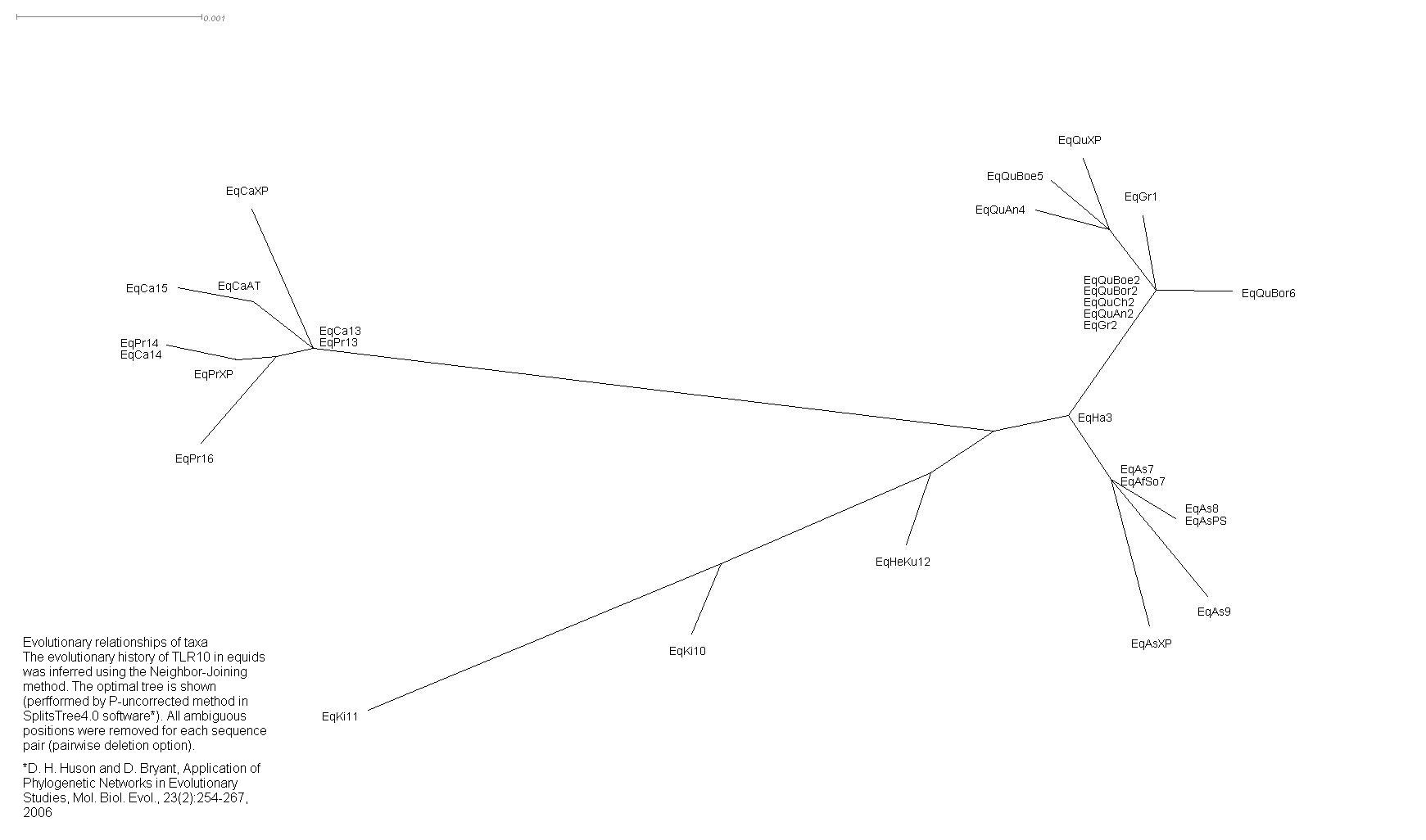

Supplement: Supplementary file 8 — Supplementary Material 8 [file 11259_2023_10245_MOESM8_ESM.zip › TLR10 NJtree equids.jpg]

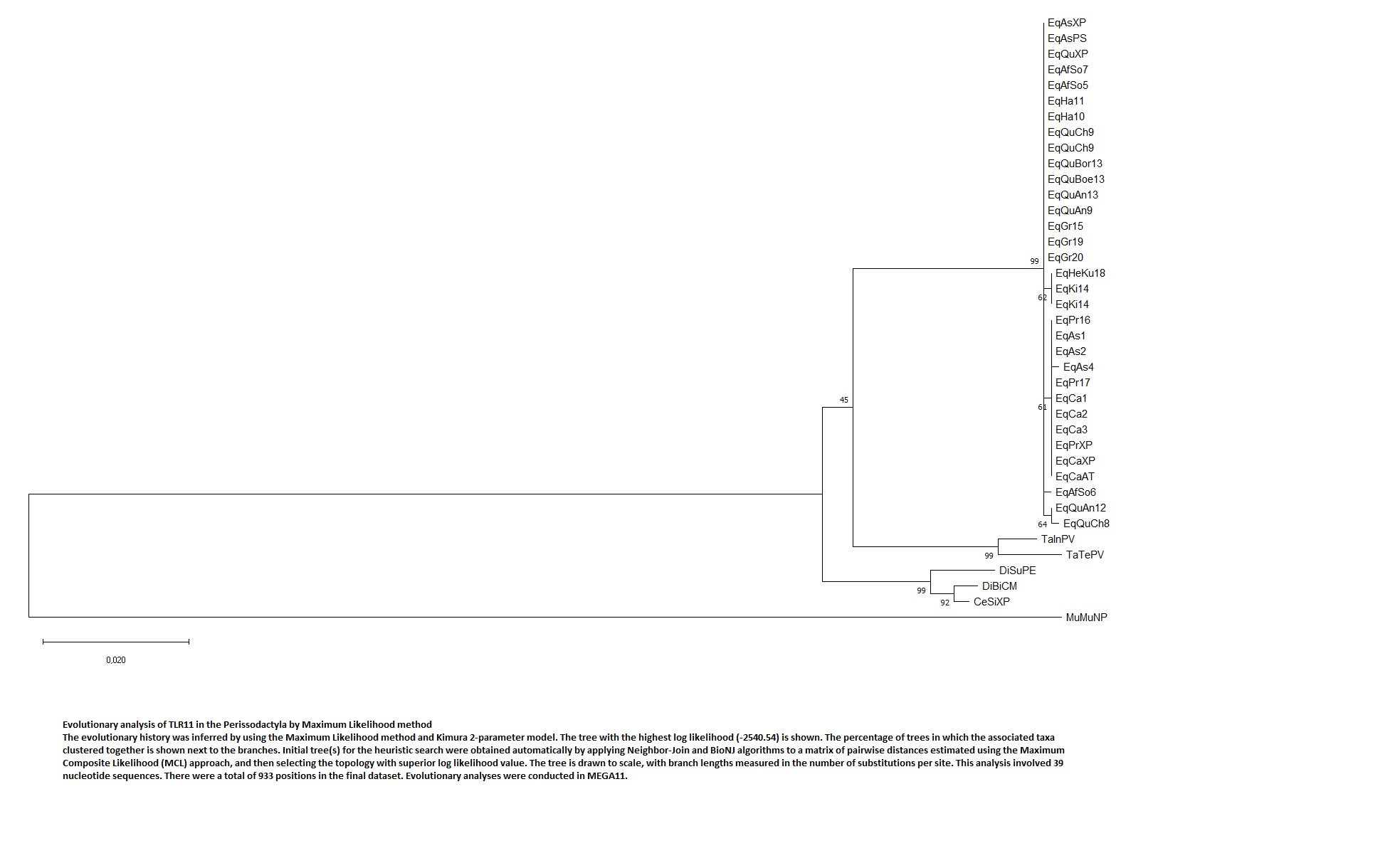

Supplement: Supplementary file 8 — Supplementary Material 8 [file 11259_2023_10245_MOESM8_ESM.zip › TLR11 ML perissodactyls.jpg]

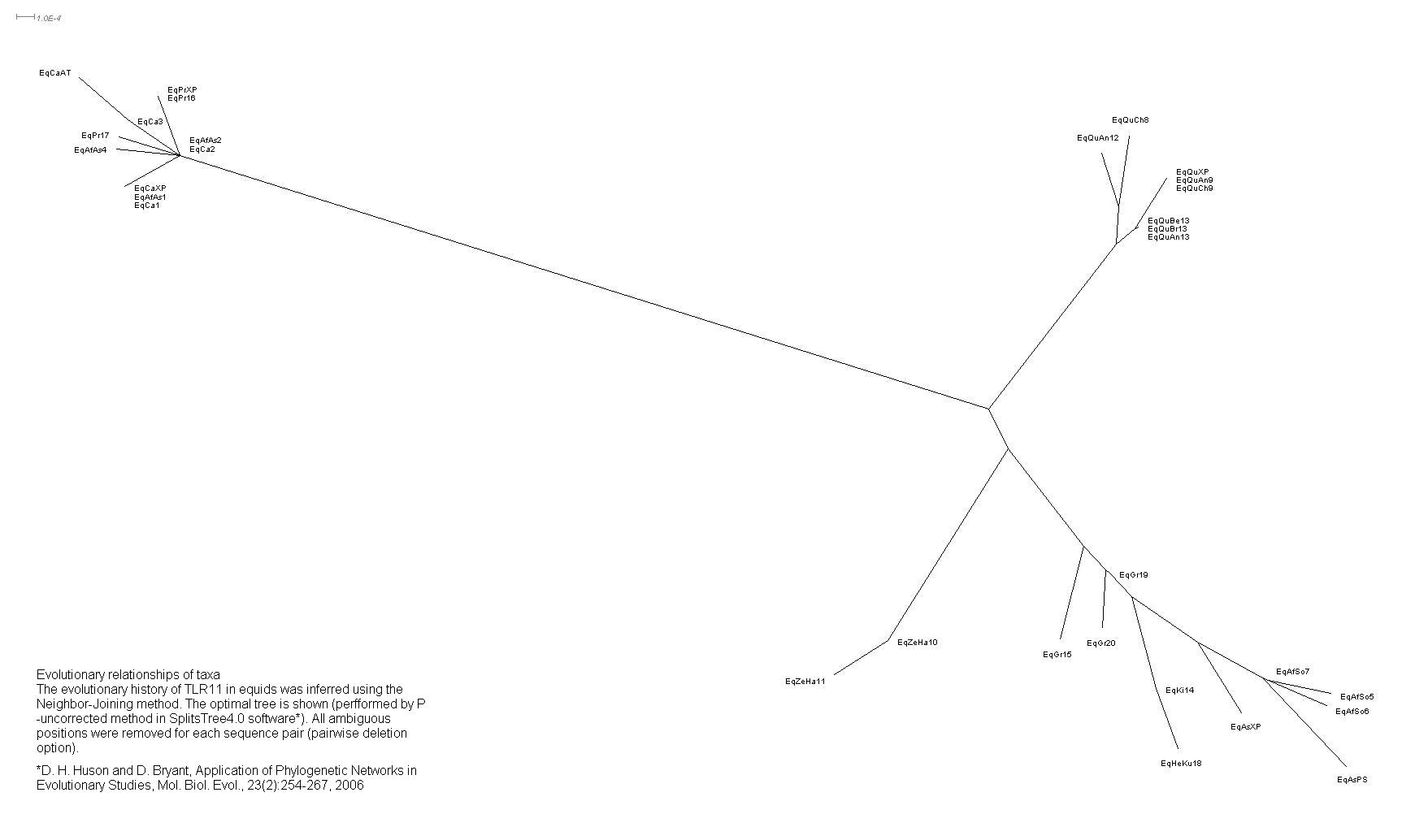

Supplement: Supplementary file 8 — Supplementary Material 8 [file 11259_2023_10245_MOESM8_ESM.zip › TLR11 NJtree equids.jpg]

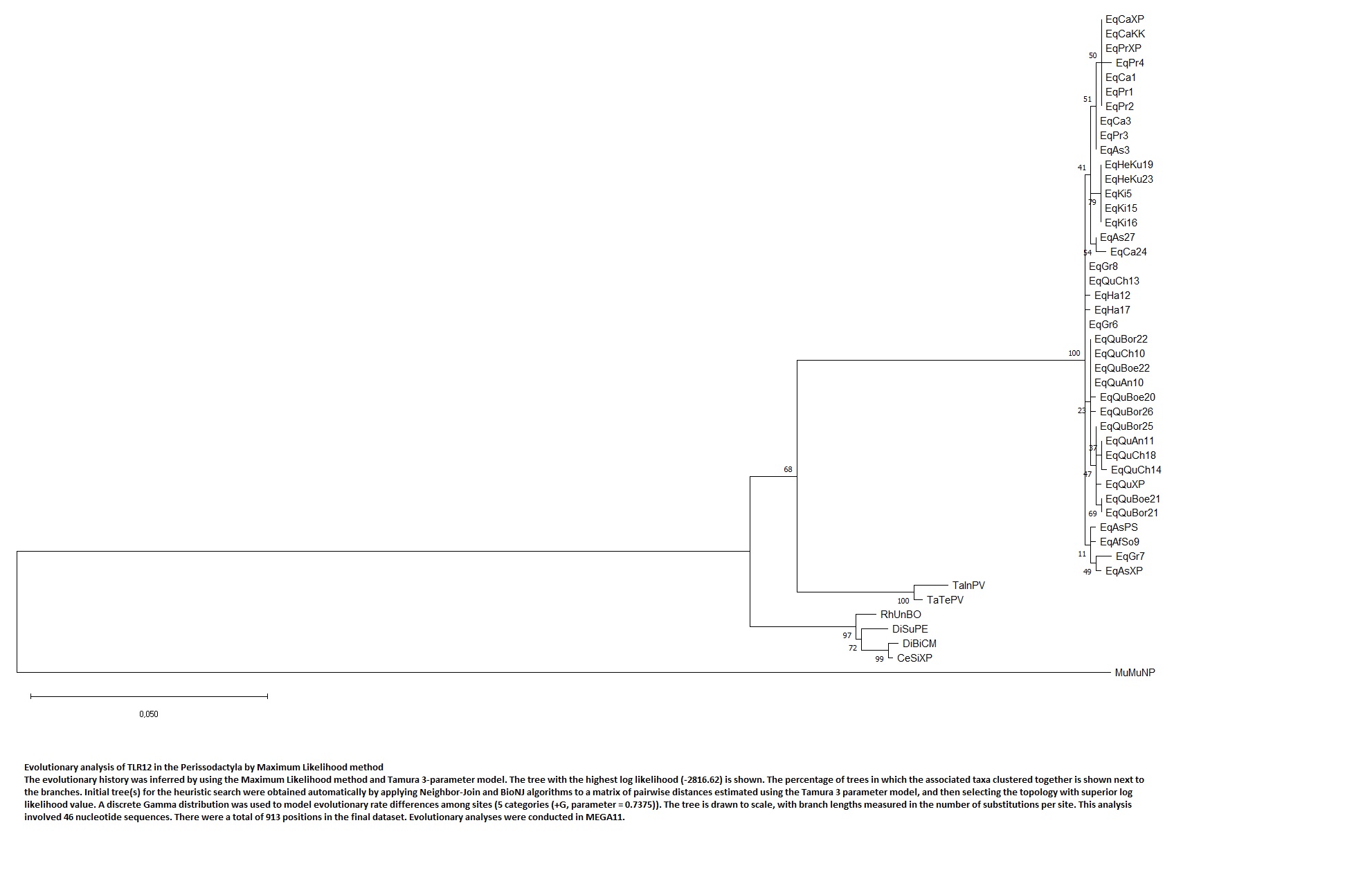

Supplement: Supplementary file 8 — Supplementary Material 8 [file 11259_2023_10245_MOESM8_ESM.zip › TLR12 ML perissodactyls.jpg]

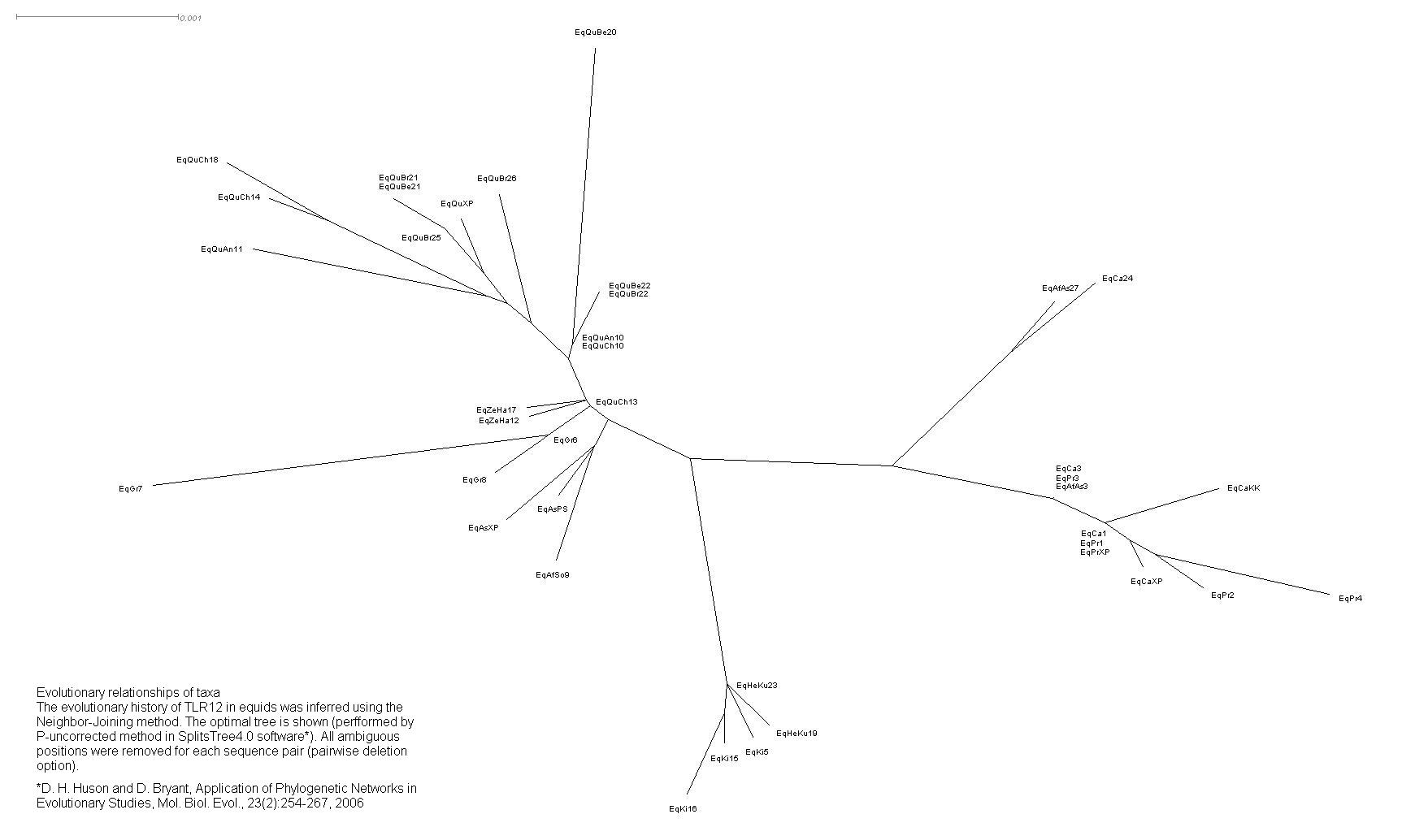

Supplement: Supplementary file 8 — Supplementary Material 8 [file 11259_2023_10245_MOESM8_ESM.zip › TLR12 NJtree equids.jpg]

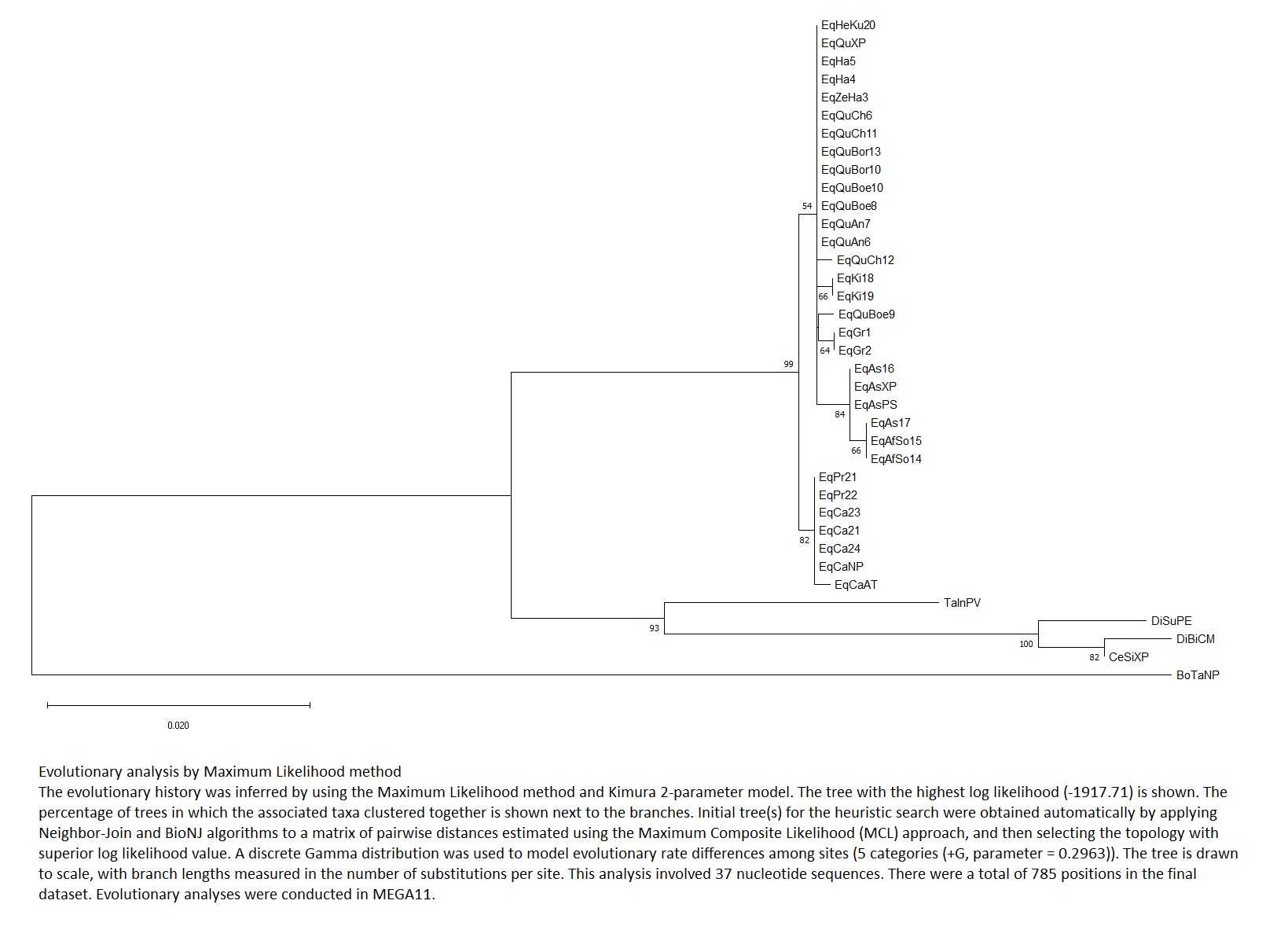

Supplement: Supplementary file 8 — Supplementary Material 8 [file 11259_2023_10245_MOESM8_ESM.zip › TLR2 ML perissodactyls.jpg]

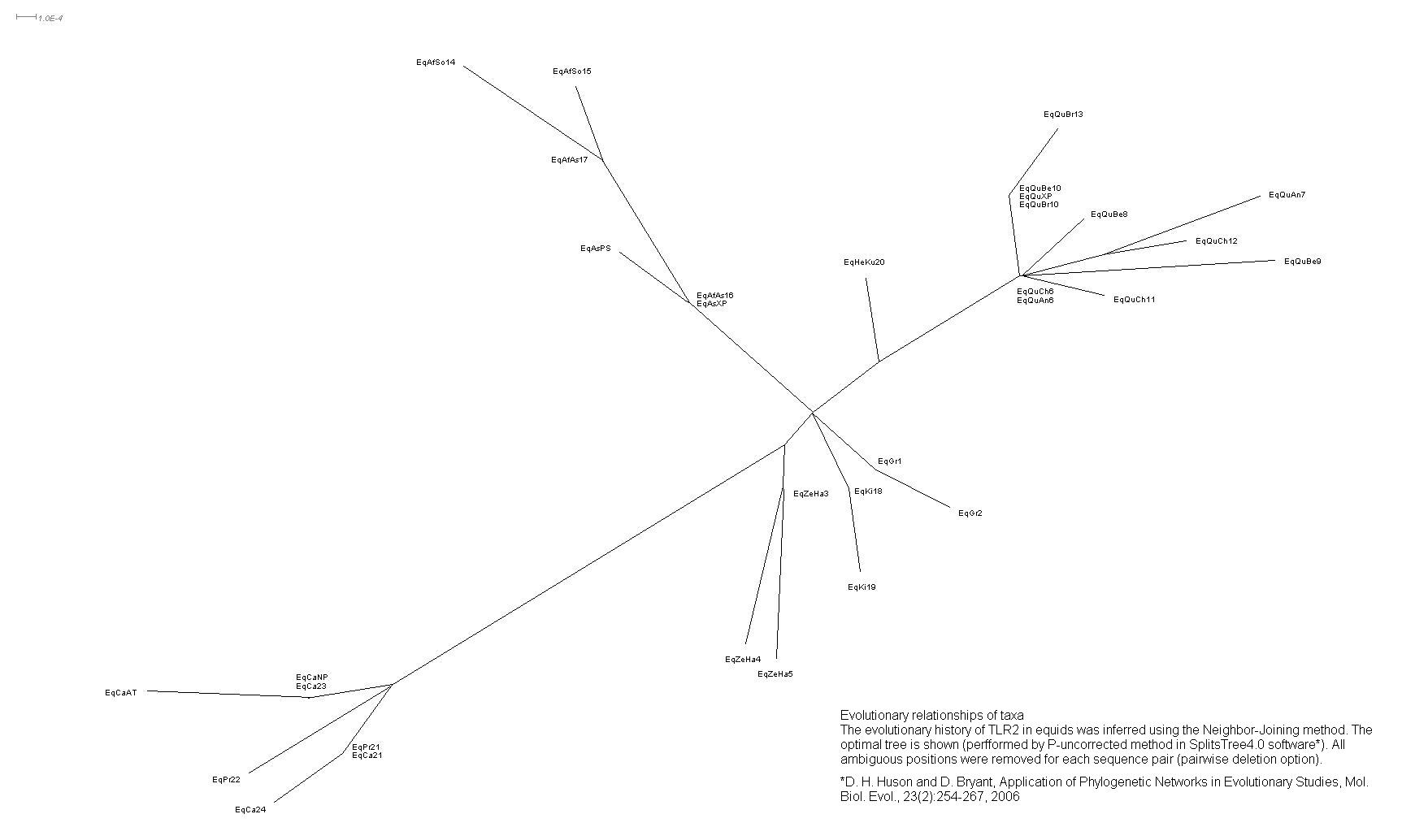

Supplement: Supplementary file 8 — Supplementary Material 8 [file 11259_2023_10245_MOESM8_ESM.zip › TLR2 NJtree equids.jpg]

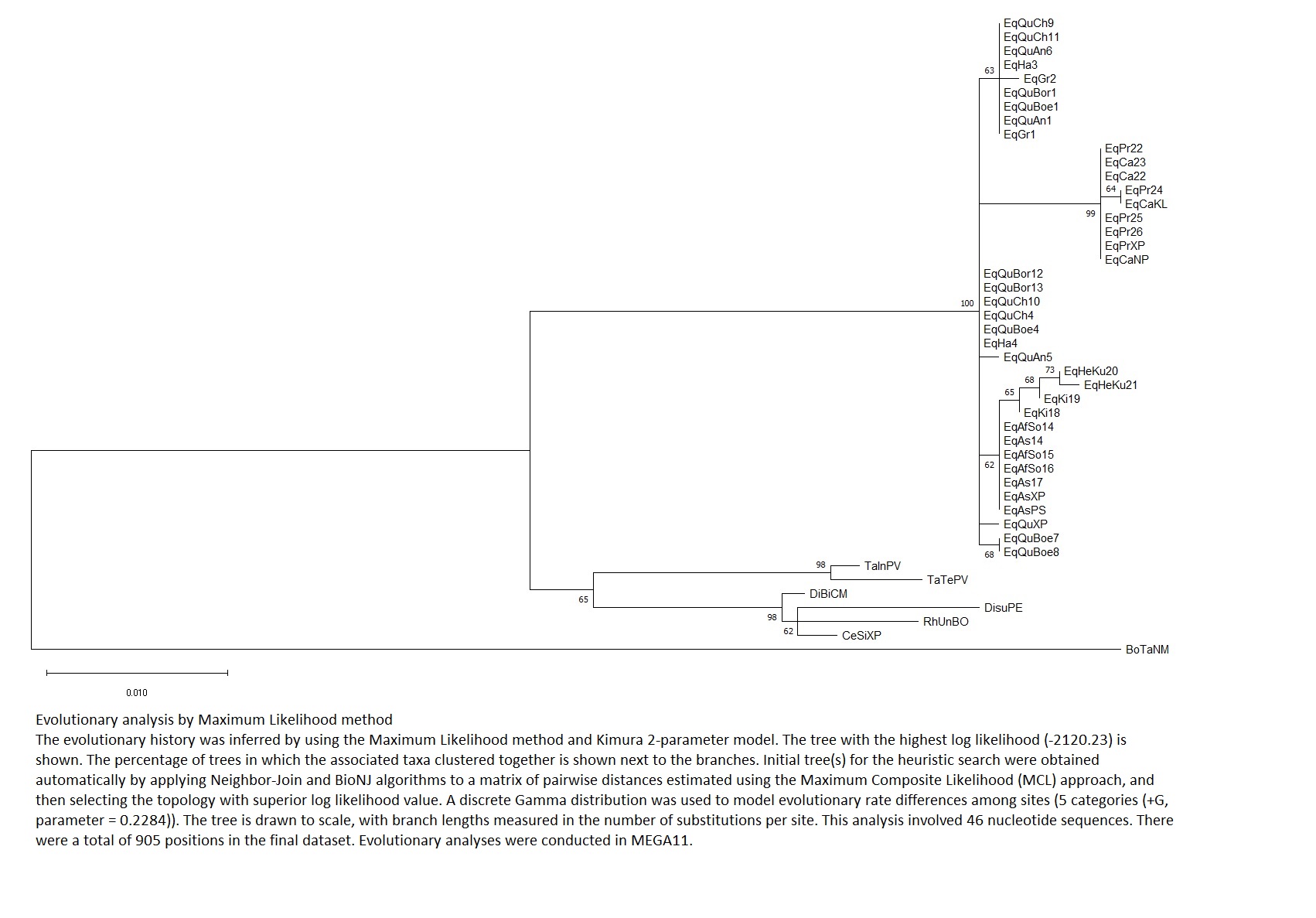

Supplement: Supplementary file 8 — Supplementary Material 8 [file 11259_2023_10245_MOESM8_ESM.zip › TLR3 ML perissodactyls.jpg]

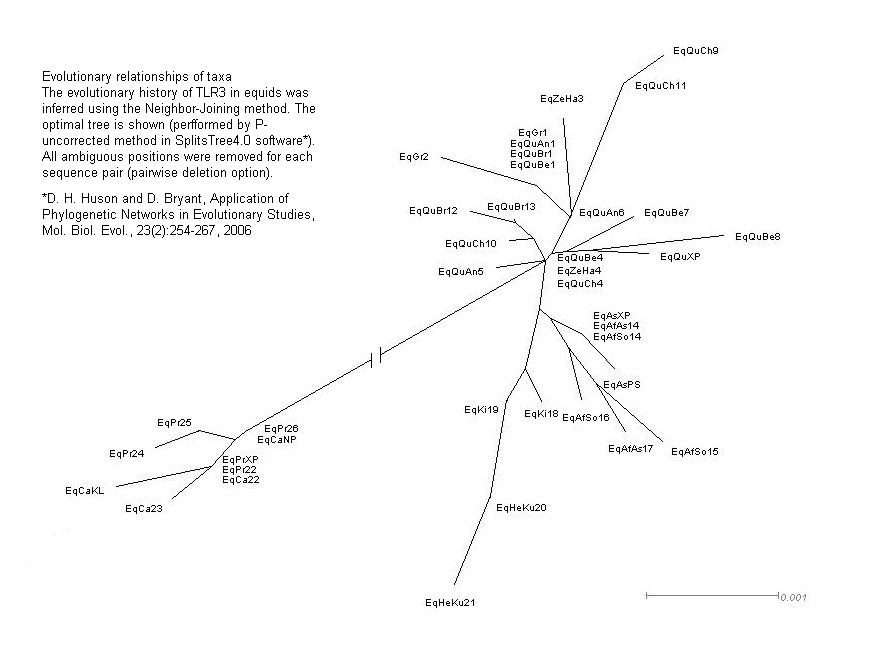

Supplement: Supplementary file 8 — Supplementary Material 8 [file 11259_2023_10245_MOESM8_ESM.zip › TLR3 NJtree equids.jpg]

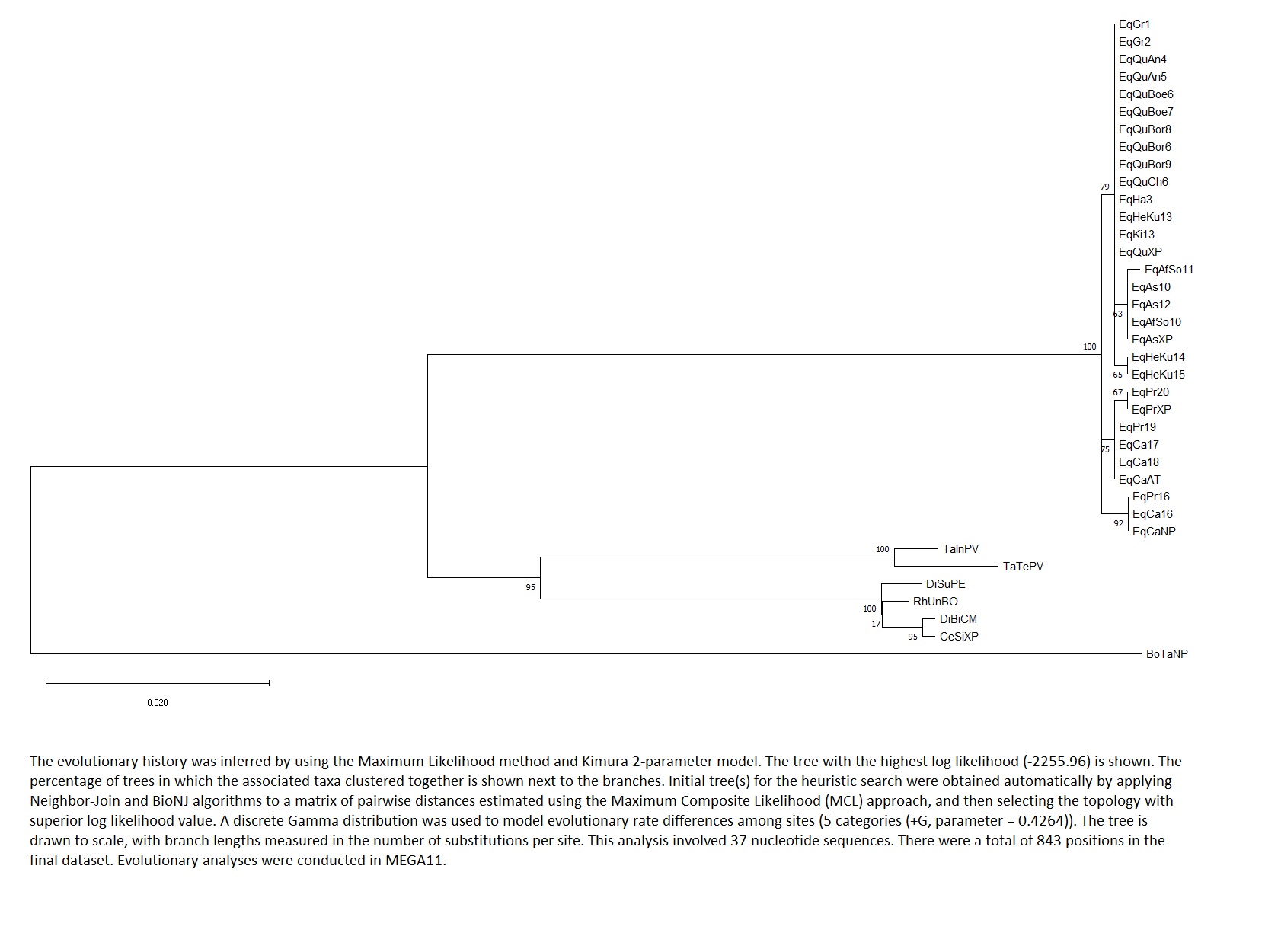

Supplement: Supplementary file 8 — Supplementary Material 8 [file 11259_2023_10245_MOESM8_ESM.zip › TLR4 ML perissodactyls.jpg]

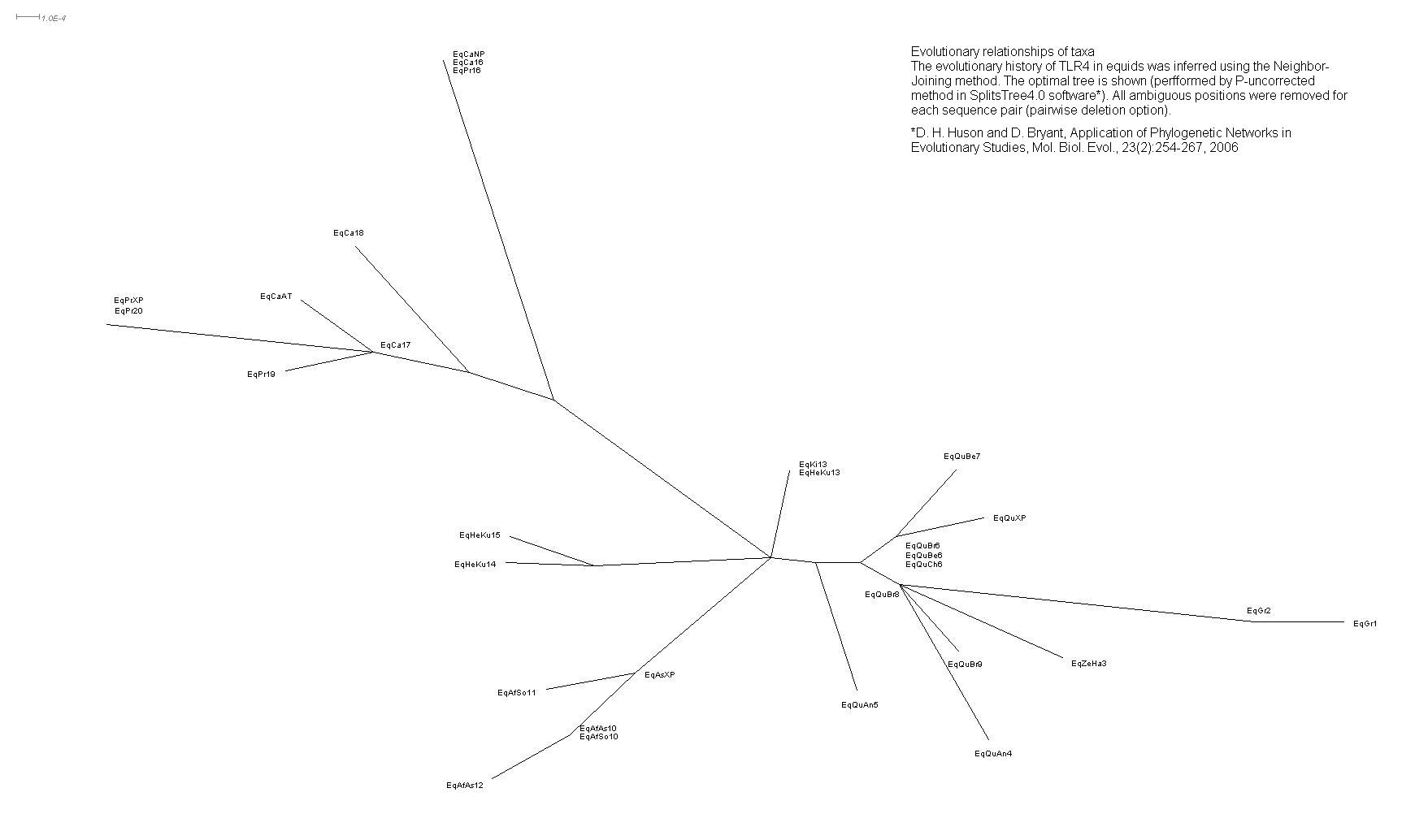

Supplement: Supplementary file 8 — Supplementary Material 8 [file 11259_2023_10245_MOESM8_ESM.zip › TLR4 NJtree equids.jpg]

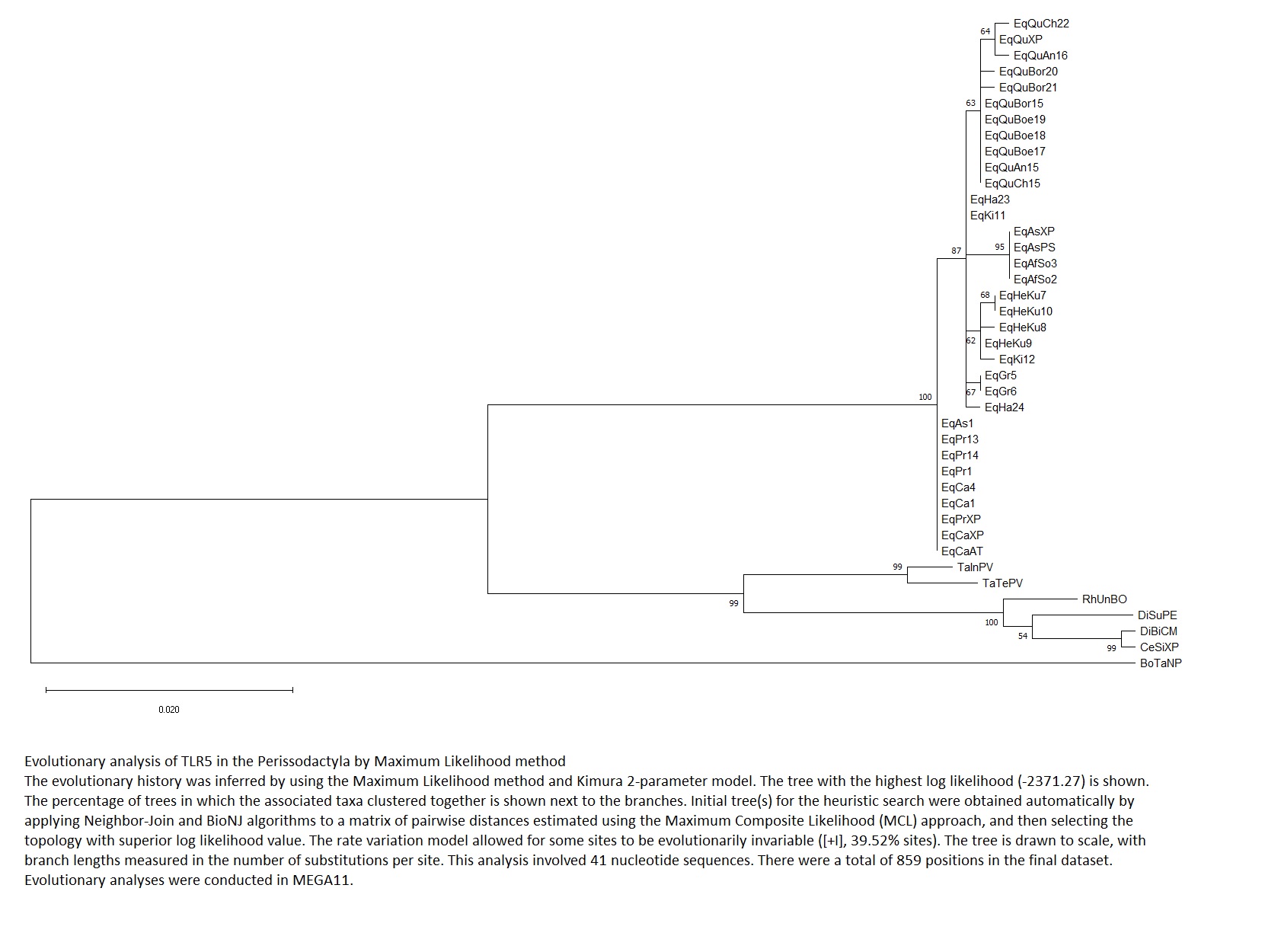

Supplement: Supplementary file 8 — Supplementary Material 8 [file 11259_2023_10245_MOESM8_ESM.zip › TLR5 ML perissodactyls.jpg]

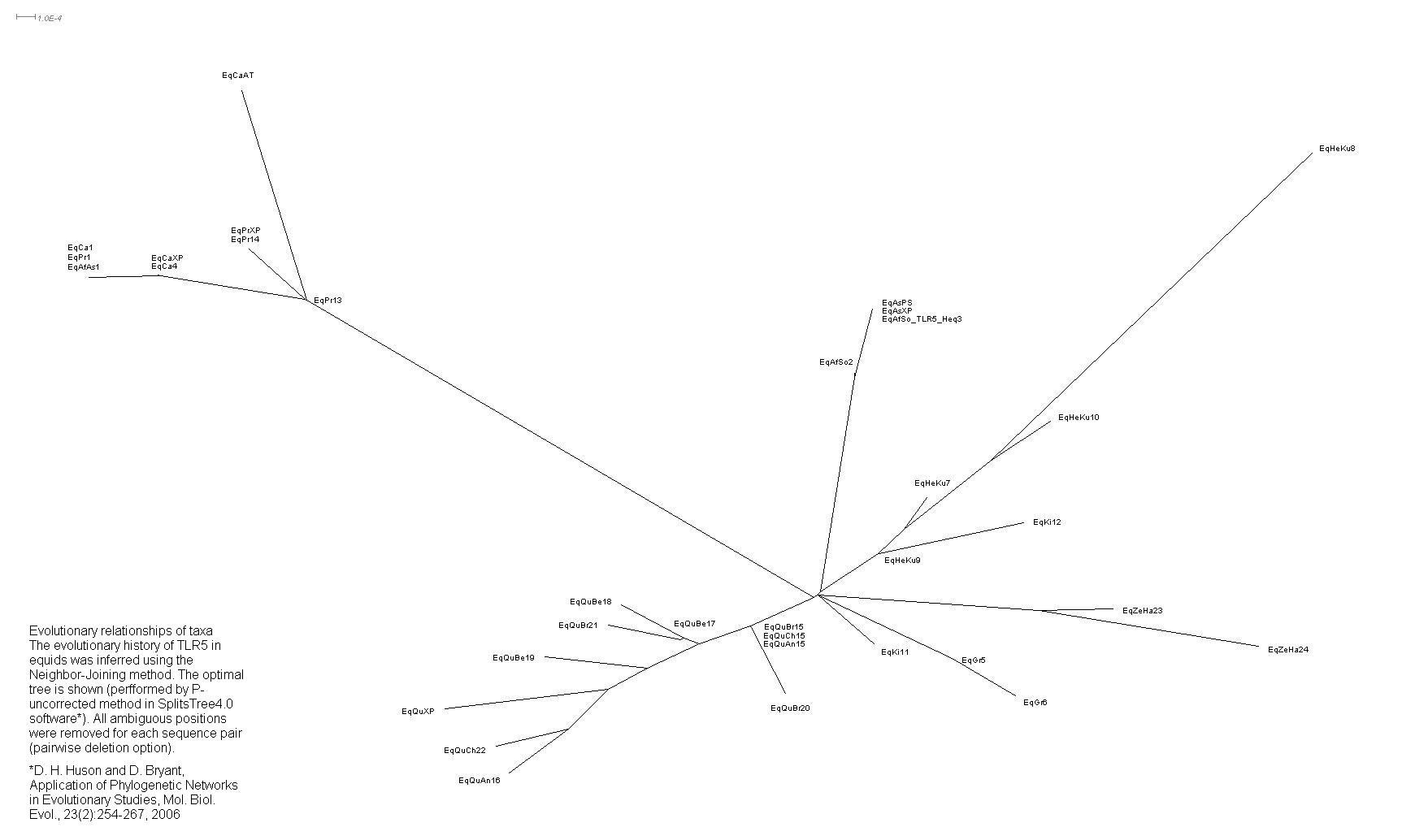

Supplement: Supplementary file 8 — Supplementary Material 8 [file 11259_2023_10245_MOESM8_ESM.zip › TLR5 NJtree equids.jpg]

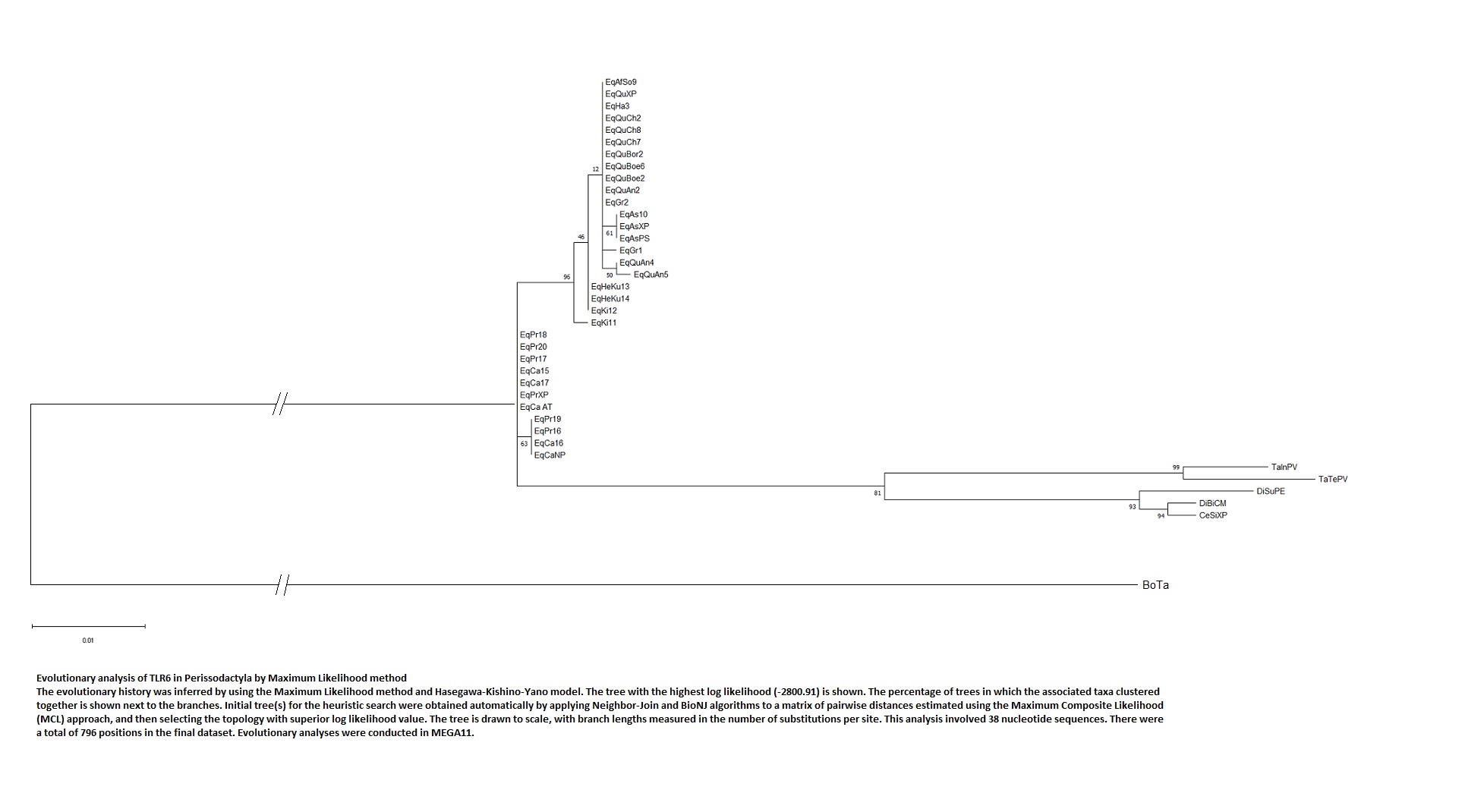

Supplement: Supplementary file 8 — Supplementary Material 8 [file 11259_2023_10245_MOESM8_ESM.zip › TLR6 ML perissodactyls.jpg]

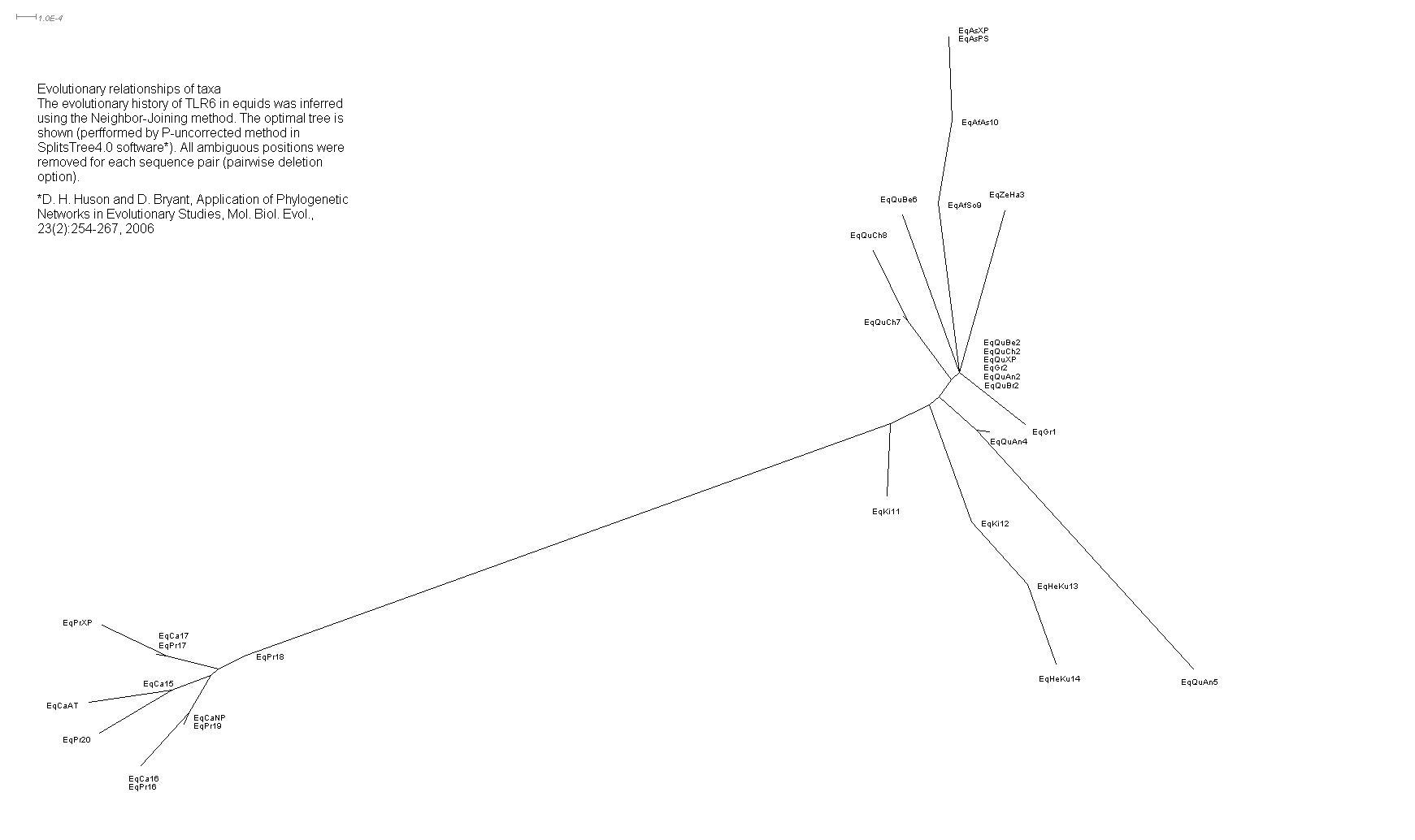

Supplement: Supplementary file 8 — Supplementary Material 8 [file 11259_2023_10245_MOESM8_ESM.zip › TLR6 NJtree equids.jpg]

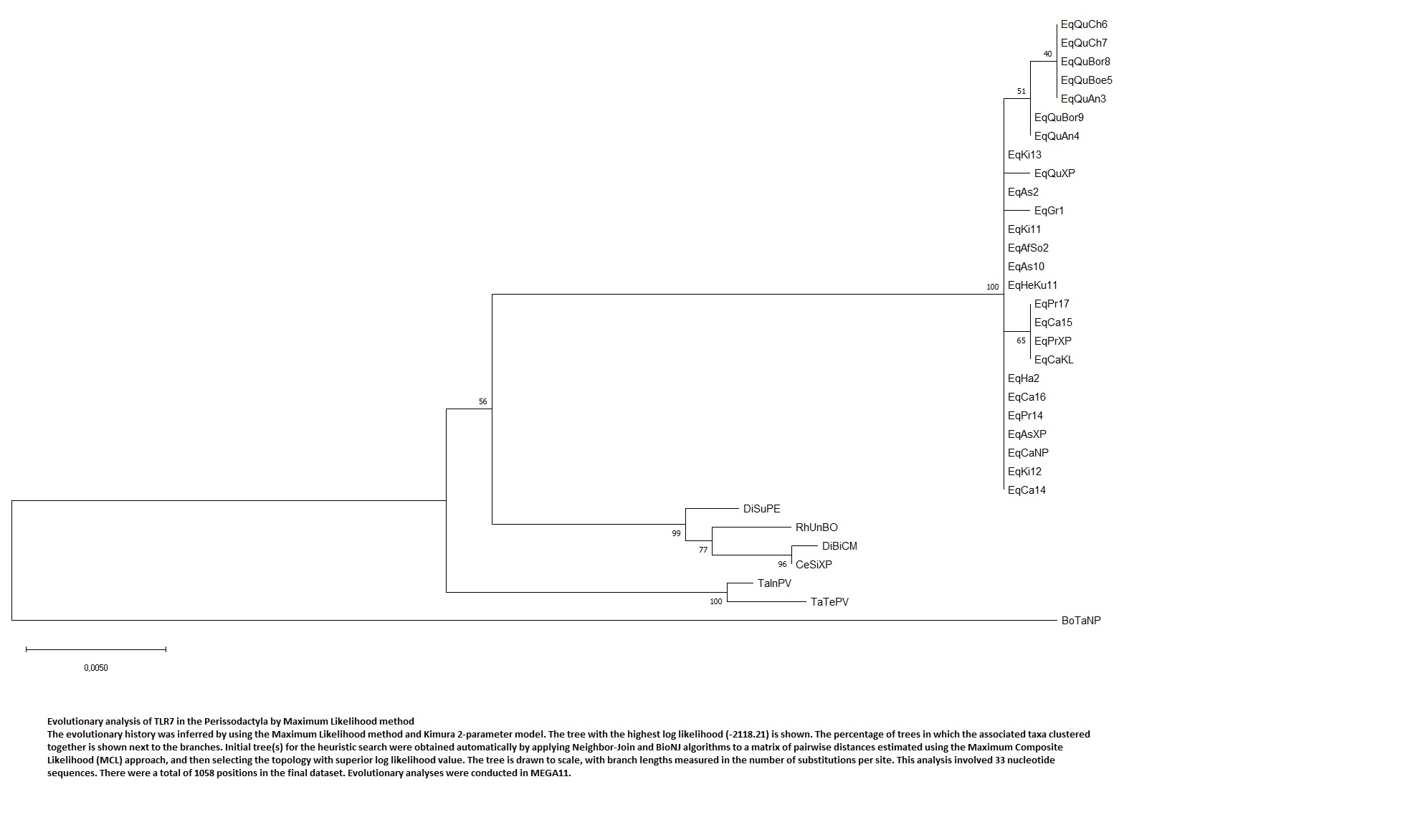

Supplement: Supplementary file 8 — Supplementary Material 8 [file 11259_2023_10245_MOESM8_ESM.zip › TLR7 ML perissodactyls.jpg]

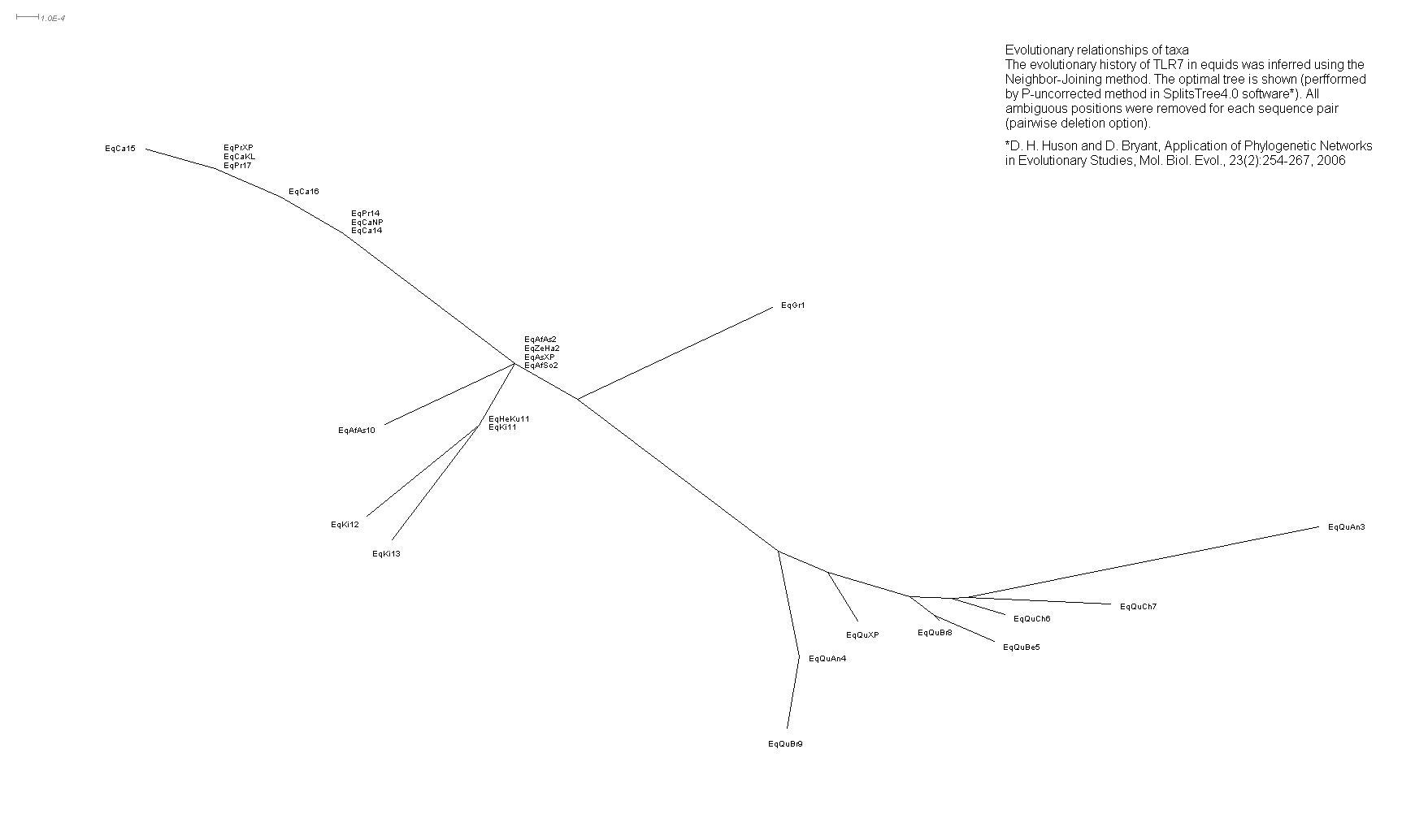

Supplement: Supplementary file 8 — Supplementary Material 8 [file 11259_2023_10245_MOESM8_ESM.zip › TLR7 NJtree equids.jpg]

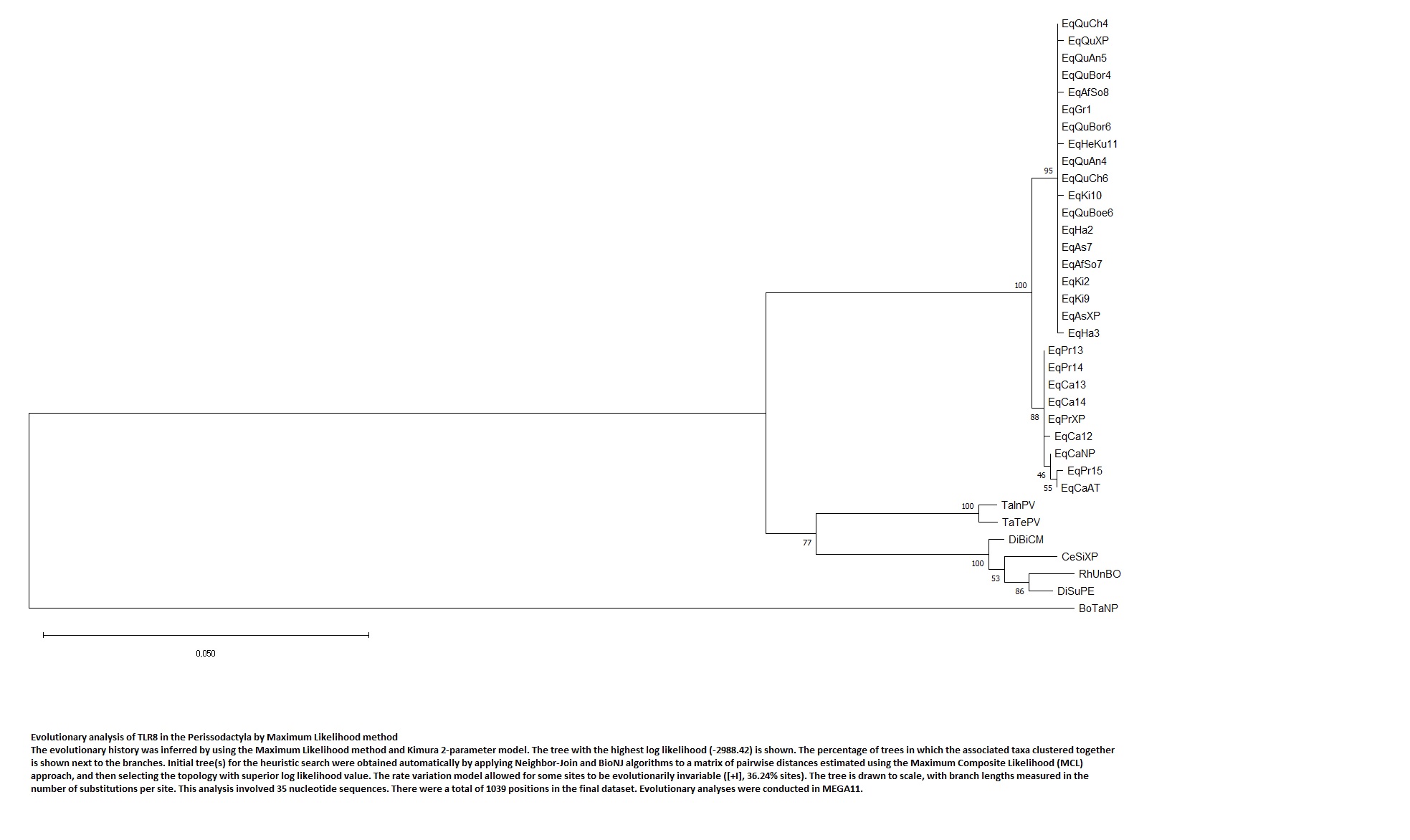

Supplement: Supplementary file 8 — Supplementary Material 8 [file 11259_2023_10245_MOESM8_ESM.zip › TLR8 ML perissodactyls.jpg]

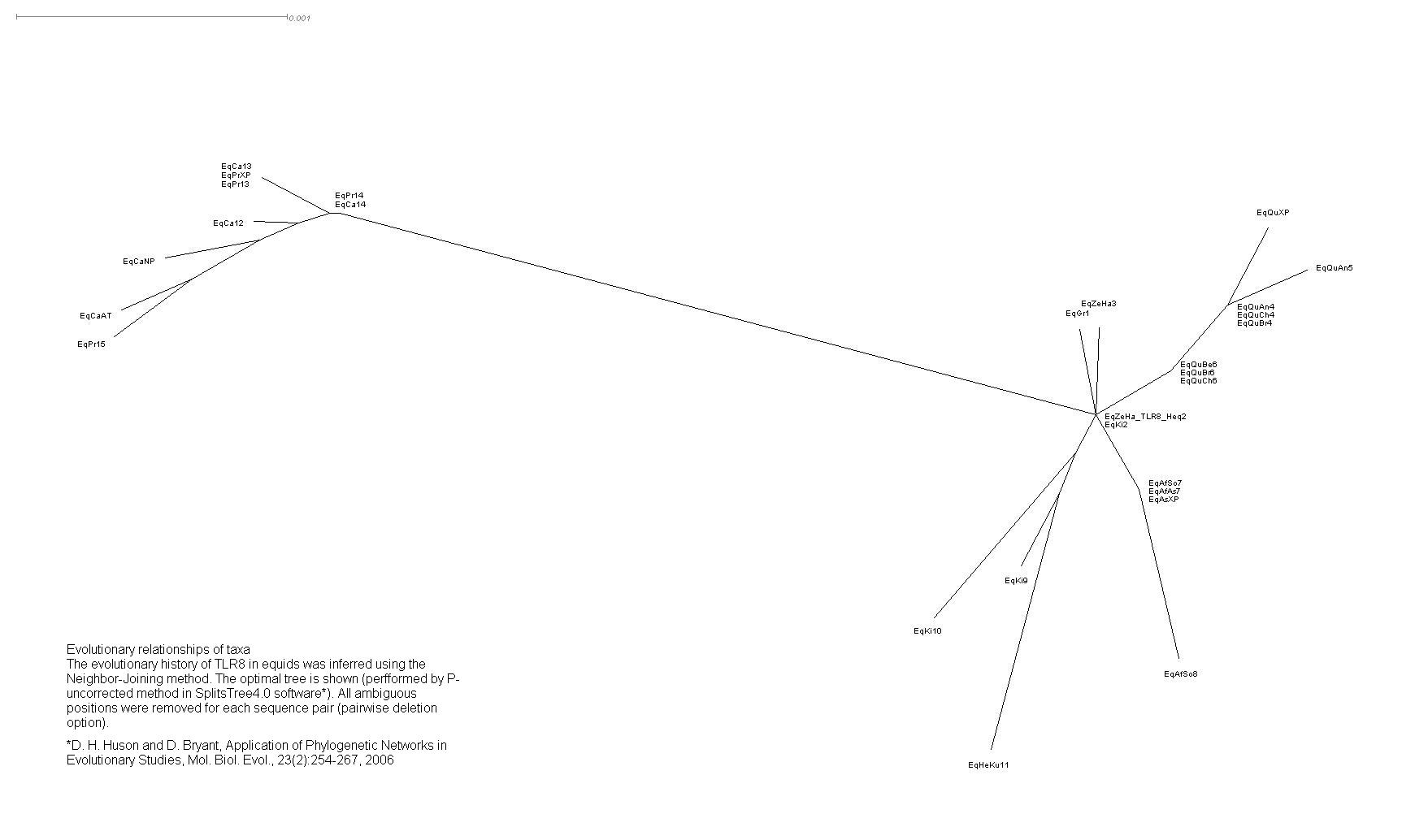

Supplement: Supplementary file 8 — Supplementary Material 8 [file 11259_2023_10245_MOESM8_ESM.zip › TLR8 NJtree equids.jpg]

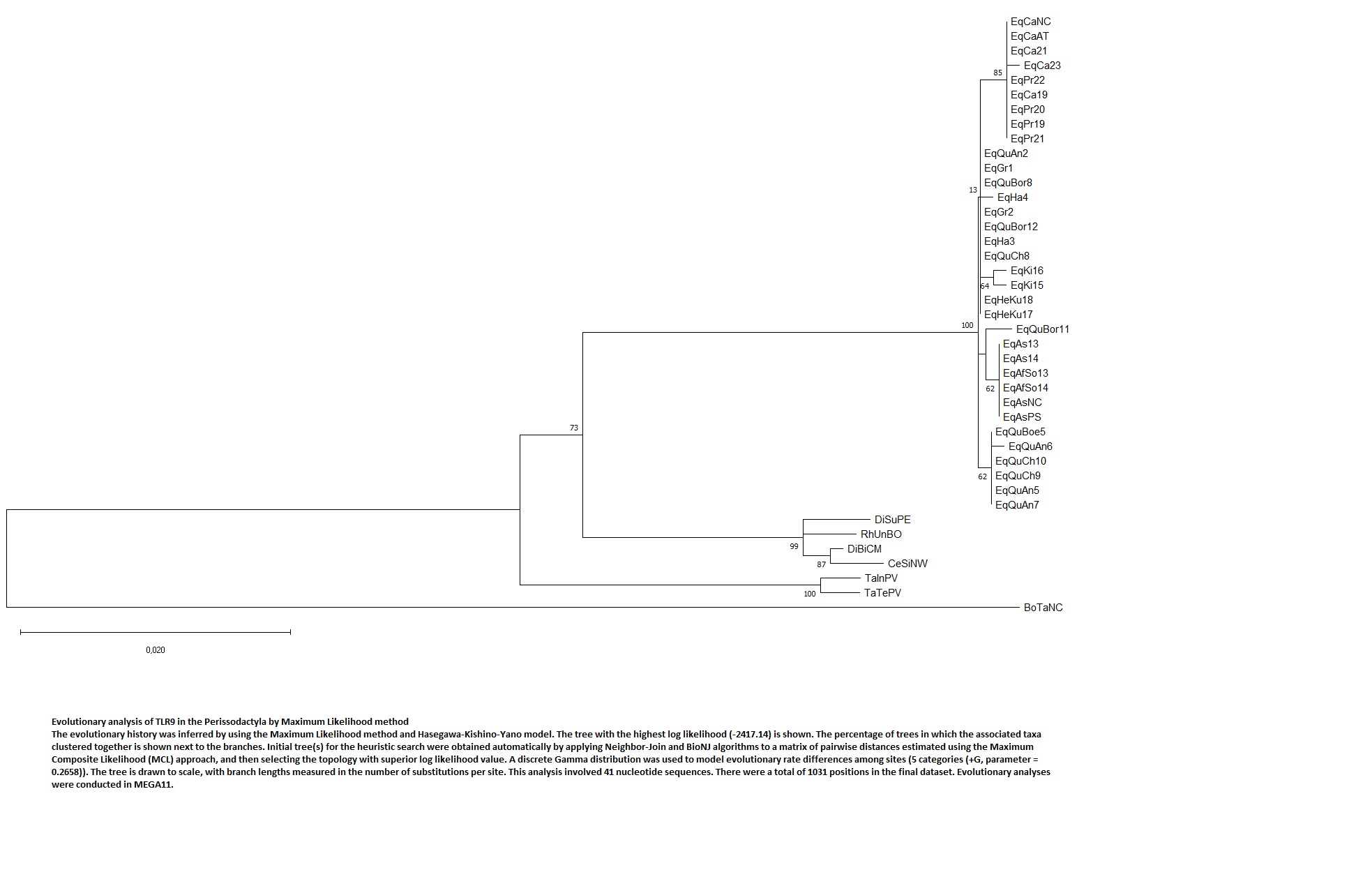

Supplement: Supplementary file 8 — Supplementary Material 8 [file 11259_2023_10245_MOESM8_ESM.zip › TLR9 ML perissodactyls.jpg]

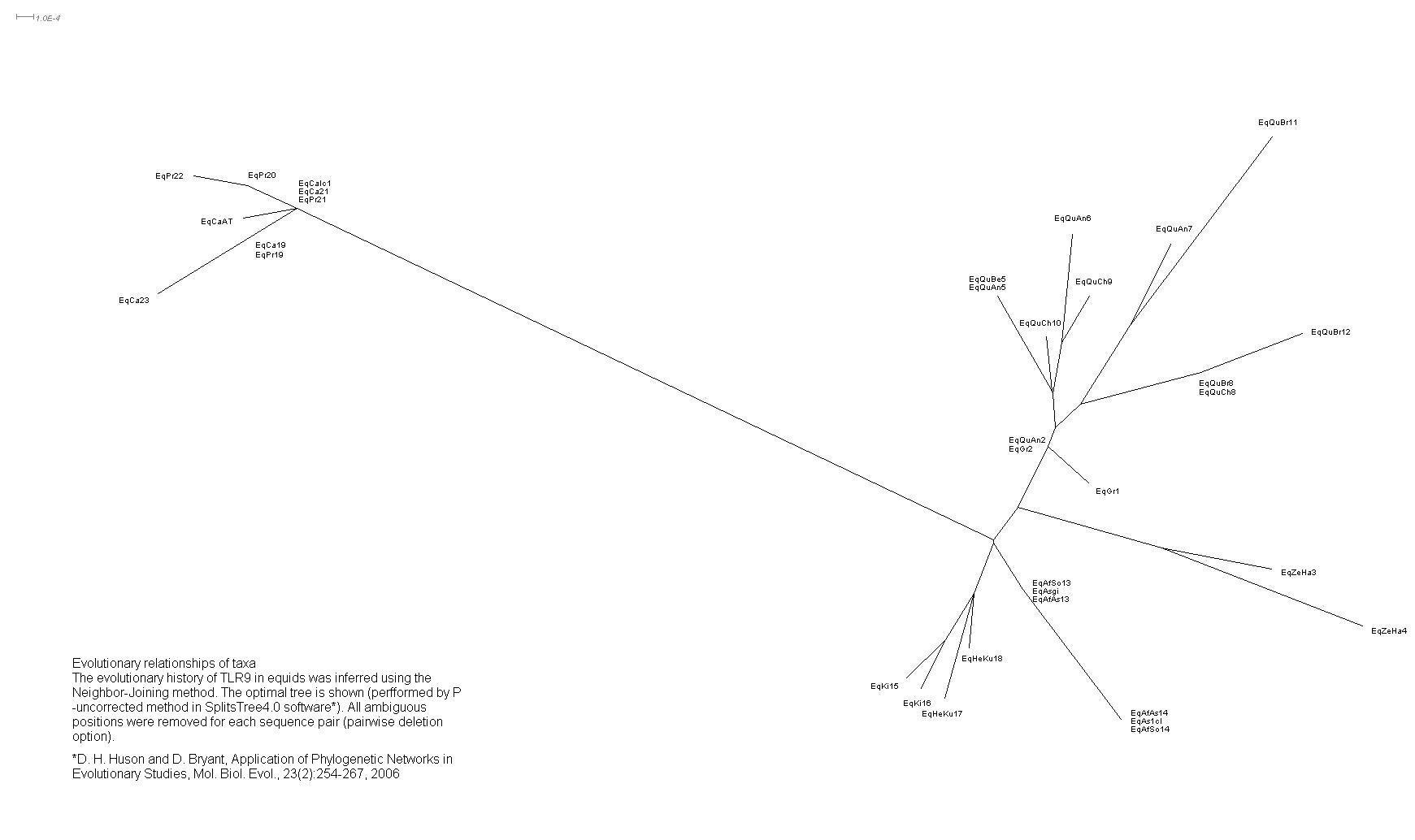

Supplement: Supplementary file 8 — Supplementary Material 8 [file 11259_2023_10245_MOESM8_ESM.zip › TLR9 NJtree equids.jpg]
